# scYOU: a multi-scale yoked optimization and unsupervised framework for analysis of cellular heterogeneity and complex-associated protein module recovery

**DOI:** 10.1093/bib/bbag626

**Published:** 2026-11-09

**Authors:** Yuan Gan, Yixiang Huang, Xinqi Gong

**Affiliations:** School of Mathematics, Renmin University of China, No. 59 Zhongguancun Street, Haidian District, 100872 Beijing, China; School of Mathematics, Renmin University of China, No. 59 Zhongguancun Street, Haidian District, 100872 Beijing, China; School of Mathematics, Renmin University of China, No. 59 Zhongguancun Street, Haidian District, 100872 Beijing, China

**Keywords:** single-cell proteomics, dual-channel framework, cellular heterogeneity, complex-associated protein module recovery, supercell-based alignment

## Abstract

Advances in high-resolution proteomic technologies are enabling systematic characterization of cellular heterogeneity and protein functional programs at increasing depth. However, existing computational approaches typically focus on either cell-level organization or protein-level structure, lacking a unified framework that jointly models both entities. Here we present scYOU, a multi-scale yoked optimization and unsupervised framework that simultaneously captures cellular heterogeneity and protein functional organization through a dual-channel neural network architecture. scYOU learns cell and protein embeddings in parallel while coupling the two views through a supercell-constrained alignment strategy. Supercells are constructed based on high-order expression similarity and define grouping structures over cells. A dual-channel alignment objective is imposed at the reconstruction level, such that reconstructed expression profiles of cells within the same supercell are encouraged to converge, whereas those from different supercells remain separable. On the protein side, Gene Ontology–derived functional similarity priors regularize protein embeddings to preserve molecular biological consistency. Across diverse benchmark datasets, scYOU achieves strong and relatively consistent clustering performance together with improved embedding separability, while the learned protein representations support embedding-guided refinement of existing interaction networks and the recovery of complex-associated protein modules. These results establish scYOU as a structurally coupled, multi-level representation learning framework for proteomic data, and highlight the importance of jointly modeling cellular heterogeneity and protein functional modules within a unified computational paradigm. Our code and data are available at https://github.com/anchor-hue/scYou.

## Introduction

With the rapid development of high-throughput sequencing and mass-spectrometry technologies, proteomics profiles at increasingly fine resolution have become widely available, enabling systematic characterization of cellular heterogeneity and functional programs. However, biological functions are rarely executed by isolated molecules; instead, they are typically orchestrated through protein–protein interaction (PPI) modules and multi-subunit protein complexes. This creates a key methodological challenge: how to learn robust representations from proteomics measurements while simultaneously recovering protein-level functional organization that is informative for downstream interaction refinement and computational recovery of complex-associated organization.

A large body of deep clustering and representation learning approaches were first developed for transcriptomics and later adopted in broader omics settings. scDeepCluster [1] jointly learns latent representations and cluster assignments by coupling an autoencoder with a clustering objective, motivated by an explicit modeling of data generation. scAce [2] employs a variational autoencoder (VAE) to learn adaptive embeddings together with clustering assignments in a unified probabilistic framework. AttentionAE-sc [3] integrates two complementary clustering strategies—one based on a ZINB-style formulation and the other based on a graph autoencoder—via an attention-fusion mechanism to obtain more stable embeddings. scMCKC [4] combines a ZINB-based autoencoder with multiple constraints (e.g. similarity-driven associations and prior pairwise constraints) and refines assignments using a weighted soft k-means procedure. scGAC (single-cell Graph Attentional Clustering [5]) leverages graph attention to learn clustering-oriented representations, denoising the neighborhood graph through attention-weighted message passing with a self-optimizing clustering strategy. In contrast, fewer methods are specifically designed for proteomics. Among these, scPROTEIN [6] explicitly models peptide-level uncertainty via multitask heteroscedastic regression and then applies graph contrastive learning to derive embeddings, followed by k-means to obtain clusters.

Such designs represent an important step toward proteomics-aware learning. However, a key limitation of existing frameworks is that they primarily operate on a single data modality, typically focusing on either cell-level representation learning or clustering-oriented embedding construction, while treating proteins implicitly or as downstream features. This design overlooks an important aspect of proteomics data: protein abundance profiles are not merely feature dimensions of cells, but instead reflect structured functional modules governed by molecular interaction and regulatory constraints.

To address this limitation, we propose scYOU, a multi-scale yoked optimization and unsupervised framework that jointly models cellular heterogeneity and protein-level functional organization from proteomics data (Fig. 1). Unlike auxiliary regularization terms commonly used in autoencoder-based methods, the protein channel is designed as an independent but coupled representation space, where protein embeddings are learned directly under gene ontology (GO)-informed functional similarity constraints. This enables the model to capture biologically meaningful relationships among proteins, such as shared functional pathways and complex-level organization, rather than treating proteins as passive observation dimensions.

**Figure 1:**
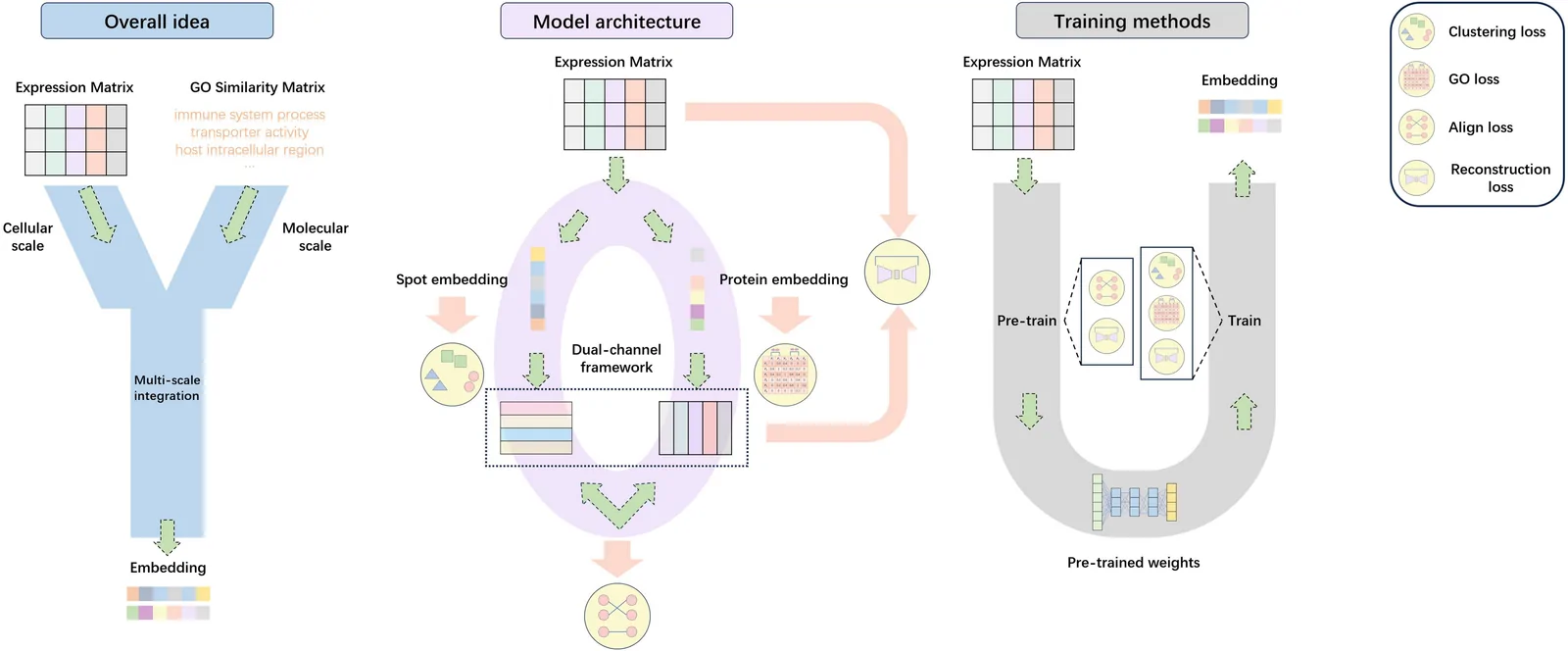
Overview of scYOU, showing (left) multi-scale integration of expression and GO similarity information, (middle) a dual-channel architecture that jointly learns cell and protein embeddings, and (right) a two-stage training strategy comprising reconstruction and alignment pretraining followed by cell clustering and GO-based protein refinement.

Crucially, this protein-level representation is not introduced as an auxiliary objective, but as a co-evolving learning channel that interacts with the cell representation space through a structured alignment mechanism. This dual-channel design enables scYOU to jointly model (i) cellular heterogeneity and (ii) protein functional organization within a unified optimization framework, providing a principled way to connect expression-level patterns with molecular interaction structure.

scYOU adopts a dual-channel architecture to learn both cell embeddings and protein embeddings simultaneously, and introduces a supercell construction strategy based on high-order expression similarity to provide adaptive alignment units. Cells without sufficiently supported local grouping are retained as singleton supercells, whereas cells with stronger local structural support form multi-cell supercells. The resulting alignment therefore combines identity-level correspondence for singleton cells with neighborhood-level cross-cell correspondence for non-singleton supercells, rather than forcing all cells into multi-cell aggregates. This design is further motivated by our prior work on multi-omics integration, where supercell-based representations were shown to be an effective intermediate-resolution abstraction for stabilizing integration and preserving biologically meaningful structure; here, we extend that validated principle to proteomics-centric representation learning. Building on supercells, scYOU applies a dual-channel alignment objective that pulls cells within the same supercell closer while separating different supercells, encouraging consistent representations across modeling perspectives. On the protein side, scYOU incorporates GO-based functional similarity priors to regularize protein embeddings, enabling embedding-guided PPI pruning that supports downstream recovery of complex-associated protein modules.

## Materials and methods

### Dual-channel architecture

scYOU adopts a dual-channel representation learning architecture composed of two multilayer perceptron (MLP)-based autoencoders, which are designed to model complementary structural information from single-cell proteomics data. One channel operates at the cell level to learn cell-centric representations from protein expression profiles, while the other captures protein-level structure to encode global relationships among proteins across cells. Each channel is trained with a reconstruction loss to preserve the intrinsic information of its input space. Although scYOU contains two representation-learning channels, it is not a conventional multi-view or multi-omics integration framework. Both channels are derived from the same single-cell proteomics expression matrix but model complementary biological entities: the cell channel represents cells according to their protein-abundance profiles, whereas the protein channel represents proteins according to their abundance patterns across cells. Consequently, the two latent spaces have asymmetric biological semantics and are not two independent views of the same observations. Their interaction is therefore implemented through alignment in the reconstructed cell-by-protein expression space rather than through direct joint embedding of multiple input modalities.

To integrate the two representation pathways, scYOU introduces an alignment loss constructed based on expression-structure-informed supercell assignments, encouraging cells within the same supercell to exhibit consistent representations across channels. In addition, a clustering loss is incorporated to directly optimize the learned embeddings toward well-separated cell clusters, and a GO similarity loss is applied to further regularize protein representations with functional biological priors.

We apply the dual-channel alignment loss in the reconstructed expression space rather than directly in the latent embedding space. This design is motivated by the asymmetric semantics of the two latent spaces: the cell channel encodes cells, whereas the protein channel encodes proteins. Thus, their latent embeddings are not directly comparable without imposing an additional mapping between different biological entities. In contrast, after decoding, both channels yield reconstructed expression representations that can be transformed into a common cell-by-protein coordinate system. Alignment in this shared reconstructed space therefore enforces cross-channel consistency at the level of observable protein abundance profiles.

This choice also preserves the functional specialization of the two latent spaces. The cell latent space remains optimized for cellular heterogeneity and clustering, whereas the protein latent space can retain protein-level organization regularized by GO similarity. The alignment objective only constrains their decoded biological outputs, allowing the two channels to share information without forcing their latent geometries to become identical.

scYOU takes processed single-cell proteomics data as input and outputs cell-level and protein-level embeddings, which are used for downstream tasks such as cell clustering and embedding-guided recovery of complex-associated protein modules. Consider a protein expression matrix $\mathbf{X} \in \mathbb{R}^{m \times n}$, where *m* and *n* denote the numbers of proteins and cells, respectively. Throughout this work, superscript indices denote row selection and subscript indices denote column selection; thus, for a matrix $\mathbf{A}$, $\mathbf{A}^{i}$ denotes its *i*th row vector and $\mathbf{A}_{j}$ its *j*th column vector.

#### Cell channel

The cell channel employs a MLP-based autoencoder to learn low-dimensional cellular embeddings while preserving critical biological variation from the expression matrix. This architecture comprises an encoder and a decoder, with its loss function combining: cell clustering loss and expression matrix reconstruction loss.

**Cell encoder**

The encoder transforms the transposed processed expression matrix $\mathbf{X}^\top \in \mathbb{R}^{n \times m}$ into a low-dimensional latent representation using a MLP architecture with progressive dimensionality reduction. Formally, let: $\mathbf{H}^{(0)}_{\textrm{cell}} = \mathbf{X}^\top $ denote the input features, $\mathbf{H}^{(k)}_{\textrm{cell}}$ represent the *k*th hidden layer features (*k = 1, … , K-1*), $\mathbf{Z}_{\textrm{cell}}$ be the output latent embedding. The hidden layer transformations are given by Equation (1):


(1) \begin{align*}& \mathbf{H}^{(k)}_{\textrm{cell}} = \sigma \left( \mathbf{H}^{(k-1)}_{\textrm{cell}} \mathbf{W}^{(k)}_{\textrm{cell}} + \mathbf{b}^{(k)}_{\textrm{cell}} \right), \quad k = 1, \dots, K-1\end{align*}

where $\mathbf{W}^{(k)}_{\textrm{cell}}$ and $\mathbf{b}^{(k)}_{\textrm{cell}}$ are learnable parameters for layer *k*, and *σ * denotes the nonlinear activation function.

The final latent embedding is computed linearly as Equation (2):


(2) \begin{align*}& \mathbf{Z}_{\textrm{cell}} = \mathbf{H}^{(K-1)}_{\textrm{cell}} \mathbf{W}^{(K)}_{\textrm{cell}} + \mathbf{b}^{(K)}_{\textrm{cell}}\end{align*}

with $\mathbf{W}^{(K)}_{\textrm{cell}}$ and $\mathbf{b}^{(K)}_{\textrm{cell}}$ being the output layer parameters.

**Cell decoder**

The decoder reconstructs high-dimensional cellular features from the latent embedding $\mathbf{Z}_{\textrm{cell}}\in \mathbb{R}^{n \times d}$ via a MLP architecture. Define the feature representations as: $\hat{\mathbf{H}}^{(0)}_{\textrm{cell}}$ (input layer), $\hat{\mathbf{H}}^{(k)}_{\textrm{cell}}$ (*k=1,… ,K-1*, hidden layers), and $\hat{\mathbf{H}}^{(K)}_{\textrm{cell}}$ (output layer). Initialized with $\hat{\mathbf{H}}^{(0)}_{\textrm{cell}} = \mathbf{Z}_{\textrm{cell}}$, hidden layers transform features nonlinearly as Equation (3):


(3) \begin{align*}& \hat{\mathbf{H}}^{(k)}_{\textrm{cell}} = \sigma \left( \hat{\mathbf{H}}^{(k-1)}_{\textrm{cell}} \hat{\mathbf{W}}^{(k)}_{\textrm{cell}} + \hat{\mathbf{b}}^{(k)}_{\textrm{cell}} \right),\quad k = 1, \dots, K-1\end{align*}

where $\hat{\mathbf{W}}^{(k)}_{\textrm{cell}}$ and $\hat{\mathbf{b}}^{(k)}_{\textrm{cell}}$ are trainable parameters and *σ * is the nonlinear activation function.

The output layer computes features linearly without activation as Equation (4):


(4) \begin{align*}& \hat{\mathbf{H}}^{(K)}_{\textrm{cell}} = \hat{\mathbf{H}}^{(K-1)}_{\textrm{cell}} \hat{\mathbf{W}}^{(K)}_{\textrm{cell}} + \hat{\mathbf{b}}^{(K)}_{\textrm{cell}}\end{align*}

with $\hat{\mathbf{W}}^{(K)}_{\textrm{cell}}$ and $\hat{\mathbf{b}}^{(K)}_{\textrm{cell}}$ denoting the final decoder parameters.

**Cell clustering loss**

Leveraging the encoder’s low-dimensional embeddings, we formulate a clustering loss by minimizing the Kullback–Leibler (KL) divergence between two probability distributions that guide cellular assignment. Specifically, given *C* cluster centroids $\{\mathbf{\mu }_{c} \in \mathbb{R}^{d}\}_{c=1}^{C}$, we first define a soft assignment probability Equation (5):


(5) \begin{align*}& q_{ic} = \frac{\left(1 + \| \mathbf{z}_{i} - \mathbf{\mu}_{c} \|^{2} / \nu\right)^{-(\nu + 1)/2}}{\sum_{c^{\prime}=1}^{C} \left(1 + \| \mathbf{z}_{i} - \mathbf{\mu}_{c^{\prime}} \|^{2} / \nu\right)^{-(\nu + 1)/2}},\end{align*}

where $\mathbf{z}_{i}$ denotes the latent embedding of the *i*th cell (*i*th row of $\mathbf{Z}_{\textrm{cell}}$), *ν * represents the Student’s *t*-distribution degrees of freedom, and $\|\cdot \|$ is the Euclidean norm.

To emphasize high-confidence assignments, we construct an auxiliary target distribution Equation (6):


(6) \begin{align*}& p_{ic} = \frac{q_{ic}^{2} / f_{c}}{\sum_{c^{\prime}=1}^{C} q_{ic^{\prime}}^{2} / f_{c^{\prime}}}, \quad f_{c} = \sum_{i=1}^{n} q_{ic}\end{align*}

where $f_{c}$ is the soft cluster frequency for category *c*.

The clustering loss is defined as the KL divergence [as shown in Equation (7)] between distributions *P* and *Q*:


(7) \begin{align*}& \mathcal{L}_{\textrm{cluster}} = \sum_{i=1}^{n} \sum_{c=1}^{C} p_{ic} \log \frac{p_{ic}}{q_{ic}}.\end{align*}

**Reconstruction loss of cell channel**

To quantify the autoencoder’s reconstruction fidelity, we define a protein expression matrix reconstruction loss as Equation (8):


(8) \begin{align*}& \tiny \mathcal{L}_{\mathrm{recon-cell}} =\frac{1}{n\cdot m} \|\mathbf{X}^\top-\hat{\mathbf{H}}^{(K)}_{\textrm{cell}}\|_{F}^{2}=\frac{1}{n\cdot m}\sum_{i=1}^{n} \sum_{j=1}^{m} \left( (\mathbf{X}^\top)_{ij}-(\hat{\mathbf{H}}^{(K)}_{\textrm{cell}})_{ij} \right)^{2},\end{align*}

where *n* and *m* denote the numbers of cells and proteins respectively, and $\|\cdot \|_{F}$ represents the Frobenius norm. This loss measures the mean squared error between the original transposed expression matrix $\mathbf{X}^\top $ and the decoder’s output $\hat{\mathbf{H}}^{(K)}_{\textrm{cell}}$.

#### Protein channel

The protein channel employs an MLP-based autoencoder comprising an encoder-decoder structure, mirroring the cell channel’s architecture. This framework optimizes two loss functions: GO similarity loss and protein expression matrix reconstruction loss.

**Protein encoder**

The protein encoder takes the processed expression matrix $\mathbf{X}\in \mathbb{R}^{m \times n}$ as input, projecting high-dimensional protein features into a low-dimensional latent space via MLP transformations. Mirroring the cell channel architecture, its encoder structure comprises Equations (9)–(11):


9 \begin{align*} & \mathbf{H}^{(0)}_{\textrm{protein}} = \mathbf{X},\end{align*}

10 \begin{align*} & \mathbf{H}^{(k)}_{\textrm{protein}} = \sigma\left(\mathbf{H}^{(k-1)}_{\textrm{protein}}\mathbf{W}^{(k)}_{\textrm{protein}} + \mathbf{b}^{(k)}_{\textrm{protein}}\right),\end{align*}

11 \begin{align*} & \mathbf{Z}_{\textrm{protein}} = \mathbf{H}^{(K-1)}_{\textrm{protein}} \mathbf{W}^{(K)}_{\textrm{protein}} + \mathbf{b}^{(K)}_{\textrm{protein}}, \end{align*}

where $\mathbf{Z}_{\textrm{protein}} \in \mathbb{R}^{m \times d^{\prime}}$ denotes the resulting low-dimensional protein embeddings.

**Protein decoder**

The protein decoder reconstructs high-dimensional protein expression features from the latent embeddings $\mathbf{Z}_{\textrm{protein}}\in \mathbb{R}^{m \times d^{\prime}}$ via MLP transformations. Identical in architecture to the cell channel decoder, it comprises Equations (12)–(14):


12 \begin{align*} & \hat{\mathbf{H}}^{(0)}_{\textrm{protein}} = \mathbf{Z}_{\textrm{protein}}, \end{align*}

13 \begin{align*} & \hat{\mathbf{H}}^{(k)}_{\textrm{protein}} = \sigma\left(\hat{\mathbf{H}}^{(k-1)}_{\textrm{protein}} \hat{\mathbf{W}}^{(k)}_{\textrm{protein}} + \hat{\mathbf{b}}^{(k)}_{\textrm{protein}}\right),\end{align*}

14 \begin{align*} & \hat{\mathbf{H}}^{(K)}_{\textrm{protein}} = \hat{\mathbf{H}}^{(K-1)}_{\textrm{protein}} \hat{\mathbf{W}}^{(K)}_{\textrm{protein}} + \hat{\mathbf{b}}^{(K)}_{\textrm{protein}}. \end{align*}

**GO similarity loss**

To leverage functional priors while preserving expression matrix information, we introduce a prior-guided regularization that aligns protein embeddings with GO semantic similarities. Let $\mathbf{S}_{\textrm{GO}} \in \mathbb{R}^{m_{\textrm{GO}} \times m_{\textrm{GO}}}$ be the predefined GO similarity matrix (see Section GO similarity matrix construction). Since GO annotations are available for most proteins but not all, we define a protein inclusion set $\mathbf{I}_{\textrm{GO}} \subset \{\textrm{protein}_{i}\}_{i=1}^{m}$ to denote the proteins with valid GO annotations:


\begin{align*} & \mathbf{I}_{\textrm{GO}}=\{\textrm{protein}_{i} \mid \textrm{protein}_{i} \textrm{ is annotated in}\ \mathbf{S}_{\textrm{GO}}\}. \end{align*}

The loss minimizes the discrepancy between embedding similarities and GO functional similarities as shown in Equation (15):


(15) \begin{align*}& \begin{split} \mathcal{L}_{\textrm{GO}} &= \frac{1}{m_{\textrm{GO}}^{2}} \sum_{\textrm{protein}_{i} \in \mathbf{I}_{\textrm{GO}}} \sum_{\textrm{protein}_{j} \in \mathbf{I}_{\textrm{GO}}} \Bigl( \mathbf{S}_{\textrm{protein}}(\textrm{protein}_{i},\textrm{protein}_{j}) \\ &\quad - \mathbf{S}_{\textrm{GO}}(\textrm{protein}_{i},\textrm{protein}_{j}) \Bigr)^{2}, \end{split}\end{align*}

where the protein embedding similarity is computed as Equation (16):


(16) \begin{align*}& \mathbf{S}_{\textrm{protein}} = \begin{bmatrix} \rho(\mathbf{Z}_{\textrm{protein}}^{i},\mathbf{Z}_{\textrm{protein}}^{j}) \end{bmatrix}_{i,j=1}^{m}, \quad \rho(\mathbf{a},\mathbf{b}) = \frac{\mathbf{a}^\top\mathbf{b}}{\|\mathbf{a}\|\|\mathbf{b}\|}.\end{align*}

Here $\mathbf{Z}_{\textrm{protein}}^{i}$ is the *i*th protein embedding vector, and $\rho (\cdot ,\cdot )$ represents the cosine similarity function.

**Reconstruction loss of protein channel**

The protein channel reconstruction loss quantifies fidelity in reproducing the original expression matrix as shown in Equation (17):


(17) \begin{align*} \tiny \mathcal{L}_{\mathrm{recon-protein}} &= \frac{1}{m\cdot n} \left\| \mathbf{X} - \hat{\mathbf{H}}^{(K)}_{\textrm{protein}} \right\|_{F}^{2}\nonumber \\ &=\frac{1}{m\cdot n}\sum_{j=1}^{m} \sum_{i=1}^{n} \left( \mathbf{X}_{ji}-(\hat{\mathbf{H}}^{(K)}_{\textrm{protein}})_{ji} \right)^{2}.\end{align*}

where *m* is the number of proteins, *n* the number of cells, and $\|\cdot \|_{F}$ the Frobenius norm. This metric computes the mean squared error between the original expression matrix $\mathbf{X}$ and the decoder’s reconstructed output $\hat{\mathbf{H}}^{(K)}_{\textrm{protein}}$.

#### Dual-channel alignment loss

Supercells group cells with consistent high-order protein expression profiles (see Section Supercell construction). To enforce cross-channel consistency, we introduce an alignment loss that operates on the reconstructed cell-wise representations produced by the two channels. Specifically, the cell channel reconstructs the expression matrix as $\hat{\mathbf{H}}^{(K)}_{\textrm{cell}} \in \mathbb{R}^{n \times m}$, while the protein channel reconstructs the matrix as $\hat{\mathbf{H}}^{(K)}_{\textrm{protein}} \in \mathbb{R}^{m \times n}$. To obtain cell-wise representations from the protein channel, we take its transpose $(\hat{\mathbf{H}}^{(K)}_{\textrm{protein}})^{\top } \in \mathbb{R}^{n \times m}$. The alignment loss therefore compares the reconstructed representation of each cell from the cell channel with the corresponding cell-wise representation induced by the protein channel.

The loss minimizes intra-supercell reconstruction discrepancies while maximizing inter-supercell separation. Formally, it is defined as


(18) \begin{align*}& \mathcal{L}_{\textrm{align}} = -\frac{1}{n} \sum_{i=1}^{n} \log \left( \frac{ \sum_{\substack{j=1 \\ I_{sp_{j}} = I_{sp_{i}}}}^{n} \exp \left( \kappa((\hat{\mathbf{H}}^{(K)}_{\textrm{cell}})^{i}, ((\hat{\mathbf{H}}^{(K)}_{\textrm{protein}})^{\top})^{j}) / \tau \right) }{ \sum_{\substack{j=1}}^{n} \exp \left( \kappa((\hat{\mathbf{H}}^{(K)}_{\textrm{cell}})^{i}, ((\hat{\mathbf{H}}^{(K)}_{\textrm{protein}})^{\top})^{j}) / \tau \right) } \right),\end{align*}

where $I_{sp_{i}}$ denotes the supercell assignment of cell *i*, $\kappa (\cdot ,\cdot )=\cos (\cdot ,\cdot )$ is the cosine similarity function, and *τ>0* is a temperature hyperparameter controlling similarity sharpness. Here $(\hat{\mathbf{H}}^{(K)}_{\textrm{cell}})^{i}$ denotes the reconstructed representation of cell *i* produced by the cell channel, while $((\hat{\mathbf{H}}^{(K)}_{\textrm{protein}})^{\top })^{j}$ denotes the reconstructed representation of cell *j* induced by the protein channel after transposing the protein reconstruction matrix.

#### Model training

The training pipeline comprises two sequential stages: pretraining followed by task-specific refinement.

**Pretraining stage**

The pretraining stage establishes fundamental cell–protein associations through joint reconstruction and cross-channel alignment objectives, as defined in Equation (19). In particular, the alignment loss $\mathcal{L}_{\textrm{align}}$ leverages supercell structure to encourage consistency between the cell-wise reconstructed representations produced by the two channels. This stage therefore provides a structured initialization that stabilizes the latent representations before downstream task-specific optimization.


(19) \begin{align*}& \mathcal{L}_{\textrm{pretrain}} = \alpha(\mathcal{L}_{\mathrm{recon-cell}} + \mathcal{L}_{\mathrm{recon-protein}}) + \beta \mathcal{L}_{\textrm{align}},\end{align*}

where $\alpha , \beta> 0$ are hyperparameters balancing reconstruction fidelity and cross-channel alignment.

**Refinement stage**

After the latent representations have been stabilized through pretraining, the model enters a task-oriented refinement stage. In this stage, the cell channel and protein channel are optimized separately using task-specific objectives, as defined in Equations (20) and (21).


(20) \begin{align*} & \mathcal{L}_{\mathrm{cell-total}} = \mathcal{L}_{\mathrm{recon-cell}} + \gamma_{\textrm{eff}} \cdot \mathcal{L}_{\textrm{cluster}},\end{align*}

21 \begin{align*} & \mathcal{L}_{\mathrm{protein-total}} = \mathcal{L}_{\mathrm{recon-protein}} + \delta \cdot \mathcal{L}_{\textrm{GO}}, \end{align*}

where *δ> 0* denotes the weight of the GO-based loss, and $\gamma _{\textrm{eff}}$ is a dynamic clustering weight defined in Equation (22):


(22) \begin{align*}& \gamma_{\textrm{eff}} = \begin{cases} \gamma, & \textrm{epoch} \leq 100, \\ \gamma \cdot \max\left(0.5, 1 - \dfrac{\textrm{epoch} - 100}{100}\right), & \textrm{epoch}> 100, \end{cases}\end{align*}

with *γ> 0* denoting the initial clustering coefficient.

During this stage, the alignment loss $\mathcal{L}_{\textrm{align}}$ is deactivated to allow the two channels to specialize under their respective task-specific objectives. The alignment objective is therefore used during pretraining to establish cross-channel correspondence, after which each channel is refined for its downstream role. The effect of retaining alignment during refinement is further evaluated in Supplementary Note S1.

The pretraining stage is terminated once the pretraining loss converges, defined as no significant decrease across successive epochs. The model then enters the refinement stage, during which the cell-level and protein-level channels are optimized independently and terminated upon convergence of their respective losses.

### GO similarity matrix construction

GO semantic similarity was calculated after mapping protein identifiers to species-specific gene annotations. For mouse and human datasets, UniProt identifiers were mapped to Entrez gene identifiers using org.Mm.eg.db and org.Hs.eg.db, respectively. Semantic similarities between annotated genes were then calculated using GOSemSim [7] for the three GO [8] branches: biological process (BP), molecular function (MF), and cellular component (CC). For proteins annotated with multiple GO terms, term-level similarities were aggregated to obtain a pairwise gene-level similarity score, which was subsequently mapped back to the corresponding protein identifiers to construct the protein–protein GO similarity matrix. During model training, we use the CC-derived similarity matrix to regularize protein embeddings, because CC directly describes subcellular localization and complex-associated CCs. BP- and MF-derived similarities were additionally examined in the extended protein-level ablation analysis provided in Supplementary Note S1.

The resulting GO similarity matrix is denoted as $\mathbf{S}_{\textrm{GO}} \in \mathbb{R}^{m_{\textrm{GO}} \times m_{\textrm{GO}}}$. Except for a small number of protein pairs whose semantic similarities cannot be computed due to missing GO annotations or identifier mappings, $\mathbf{S}_{\textrm{GO}}$ covers the semantic similarities for nearly all protein pairs in the expression matrix $\mathbf{X}$.

### Supercell construction

Supercells define adaptive alignment units from local high-order protein-expression structure, following our previously published strategy [9]. In scYOU, supercells serve as weak local-structure constraints for representation learning rather than final clustering labels. At the dual-channel autoencoder’s output layer, we introduce a supercell-guided alignment loss that attracts cells within the same supercell while repelling cells from distinct supercells.

The construction is based on Pearson-correlation neighborhoods of the protein expression matrix, and cells not retained during stochastic pruning are preserved as singleton supercells. Consequently, singleton supercells retain identity-level cross-channel correspondence, whereas non-singleton supercells additionally introduce cross-cell positive relationships among locally supported cells. The supercell construction proceeds as follows:

We compute pairwise cell similarity using the Pearson correlation coefficient (PCC) on mean-centered expression profiles. For cell vectors $\mathbf{x}_{i},\mathbf{x}_{j} \in \mathbb{R}^{m}$, let $\tilde{\mathbf{x}}_{i}=\mathbf{x}_{i}-\bar{x}_{i}\mathbf{1}$ and $\tilde{\mathbf{x}}_{j}=\mathbf{x}_{j}-\bar{x}_{j}\mathbf{1}$, where $\bar{x}_{i}$ and $\bar{x}_{j}$ are the corresponding mean expression levels. The similarity is defined as


(23) \begin{align*}& s_{ij}=\rho(\mathbf{x}_{i},\mathbf{x}_{j})= \frac{\tilde{\mathbf{x}}_{i}^\top \tilde{\mathbf{x}}_{j}} {\|\tilde{\mathbf{x}}_{i}\|_{2}\,\|\tilde{\mathbf{x}}_{j}\|_{2}},\end{align*}

yielding the similarity matrix $\mathbf{S}\in \mathbb{R}^{n\times n}$.

We introduce a second-order co-occurrence neighborhood matrix $\mathbf{N} \in \{0,1\}^{n \times n}$ to capture higher-order cellular relationships. For cell *i*, denote its *a*-nearest neighbor set as $\mathcal{N}_{i}^{a}$. The matrix entries are defined by the shared neighborhood criterion Equation (24):


(24) \begin{align*}& n_{ij} = \begin{cases} 1, & \textrm{if}\ j \in \mathcal{N}_{i}^{a} \textrm{ and}\ |\mathcal{N}_{i}^{a} \cap \mathcal{N}_{j}^{a}| \geq b, \\ 0, & \textrm{otherwise}. \end{cases}\end{align*}

where *a* (neighborhood size) and *b* (shared neighbor threshold) are hyperparameters.

We construct a second-order similarity graph $\mathcal{G}_{N}$ using the adjacency matrix $\mathbf{N}$, decomposing it into connected components $\{\mathcal{G}_{N}(1), \dots , \mathcal{G}_{N}(L)\}$. Cells within each component exhibit homogeneous protein expression and form cohesive groups. However, peripheral cells showing weak graph connectivity require targeted removal.

We quantify the structural importance of each cell within connected components via degree centrality. For a cell *i* in component $\mathcal{G}_{N}(l)$, its centrality is normalized as Equation (25):


(25) \begin{align*}& \mathbf{DC}_{i}{(l)} = \frac{\deg(i)}{|\mathcal{G}_{N}(l)|-1}, i\in \mathcal{G}_{N}(l)\end{align*}

where *\deg (i)* denotes the node degree within the connected component, and $|\mathcal{G}_{N}(l)|$ represents the vertex count of $\mathcal{G}_{N}(l)$.

Cells with high structural centrality tend to share greater protein expression similarity with their component’s global profile, enhancing their retention likelihood. We define the retention probability as Equation (26):


(26) \begin{align*}& \mathbf{RP}_{i}{(l)} = \begin{cases} \frac{\mathbf{DC}_{i}(l)-\underset{j}\min{\mathbf{DC}_{j}(l)}}{\underset{j}\max{\mathbf{DC}_{j}(l)}-\underset{j}\min{\mathbf{DC}_{j}(l)}}, &\! \textrm{if}\ i \in \mathcal{G}_{N}(l) \textrm{ and}\ \underset{j}\max{\mathbf{DC}_{j}(l)}\neq\underset{j}\min{\mathbf{DC}_{j}(l)}, \\ 1, &\! \mathrm{otherwise.} \end{cases}\end{align*}

Let $\mathbf{r} \in \{0,1\}^{n}$ denote the cell removal indicator vector, where $r_{i} = 1$ if and only if cell *i* is eliminated. The removal decision follows a Bernoulli distribution as Equation (27):


(27) \begin{align*}& r_{i} \sim \textrm{Bernoulli}(1 - \mathbf{RP}_{i}{(l)}) \quad \textrm{for} \quad i \in \mathcal{G}_{N}{(l)}.\end{align*}

Each resulting entity—whether a pruned connected component or an individual eliminated cell—constitutes a *supercell*. The supercell membership mapping $\mathbf{I_{sp}} \in \mathbb{Z}^{n}_{+}$ assigns each cell to its corresponding supercell index.

To further assess whether the stochastic construction introduces dataset-specific bias, we report the number of supercells, supercell size distributions, singleton-cell fractions, and seed-wise robustness analyses in Supplementary Note S1.

### Datasets and preprocessing

To benchmark our method, we evaluated its performance on six publicly available single-cell proteomics datasets generated using high-throughput mass spectrometry (MS), using the processed protein-level abundance matrices released by the corresponding studies (Table 1).

(1) **Montalvo *et al.*** [10]: The Montalvo dataset was generated using the SCoPE2 workflow with nPOP-based sample preparation, quantifying 501 proteins across 843 cells derived from stem cell–differentiated pancreatic lineages, including Beta1, Beta2, Alpha, Delta, and Vasculature cell populations.
(2) **Specht *et al.*** [11]: The Specht dataset was prepared using the SCoPE2 method. It quantifies 3042 proteins across 1490 single cells, comprising two cell types: monocytes (sc_u) and macrophage-like cells (sc_m0).
(3) **Khan *et al.*** [12]: The Khan dataset was generated using the SCoPE2, pSCoPE, and plexDIA workflows with nPOP-based sample preparation. It quantifies 3178 proteins across 1408 single cells from human testis, encompassing six cell types: endothelial cells, peritubular myoid cells, Leydig cells, spermatogonia, spermatocytes, and spermatids.
(4) **Huffman *et al.*** [13]: The Huffman dataset was generated using the pSCoPE workflow, quantifying 1123 proteins across 373 single primary macrophages under two experimental conditions: untreated and lipopolysaccharide–stimulated.
(5) **Leduc *et al.* (plexDIA)** [14]: The Leduc-plexDIA dataset was generated using the nPOP workflow for nano-proteomic sample preparation, quantifying 4619 proteins across 121 single cells derived from three distinct cellular sources, including monocytes, pancreatic ductal adenocarcinoma (PDAC), and melanoma cells.
(6) **Leduc *et al.* (TMT32)** [14]: The Leduc-TMT32 dataset was generated using the nPOP workflow for nano-proteomic sample preparation, quantifying 1775 proteins across 1526 single cells encompassing monocyte, PDAC, and melanoma populations.

**Table 1:** Detail information of six proteomics datasets.

Datasets	#Cell	#Protein	#Type	Refs
Montalvo	843	501	5	Ref [10]
Specht	1490	3042	2	Ref [11]
Khan	1408	3178	6	Ref [12]
Huffman	373	1123	2	Ref [13]
Leduc-plexDIA	121	4619	3	Ref [14]
Leduc-TMT32	1526	1775	3	Ref [14]

For all six datasets, we used the processed protein-level abundance matrices released by the original studies rather than reprocessing raw MS data. Protein identification, quantification, and primary quality-control procedures therefore followed the corresponding studies, and only the additional preprocessing required by our clustering pipeline was applied thereafter.

### Cell clustering compared methods

Due to the limited availability of computational methods specifically designed for single-cell proteomics data, we compared scYOU with three proteomics-oriented representation or clustering methods and five state-of-the-art clustering methods originally developed for single-cell transcriptomics.

**scPROTEIN [6]**: scPROTEIN is a versatile framework designed for single-cell proteomics data analysis, which consists of peptide uncertainty estimation based on a multitask heteroscedastic regression model and cell embedding generation based on graph contrastive learning. We perform k-means clustering on this embedded feature to obtain the cell clustering results.

**scPGCL [15]**: scPGCL is a graph contrastive learning framework specifically designed for clustering MS-based single-cell proteomics data. It adaptively reconstructs intercellular relationship graphs through gradient-guided structure learning and employs a gradient-weighted contrastive loss to reduce the influence of false-negative samples, thereby preserving relationships among biologically similar cells and learning clustering-oriented cell representations.

**omicsGMF [16]**: omicsGMF is a scalable generalized matrix factorization framework developed for dimensionality reduction of bulk and single-cell proteomics data. It jointly models latent sample and protein factors while accommodating missing values and known sample- or feature-level covariates, enabling dimensionality reduction and correction of technical variation within a unified framework. We perform k-means clustering on the low-dimensional sample representation learned by omicsGMF to obtain the cell clustering results.

**scDeepCluster [1]**: scDeepCluster is a single-cell model-based deep embedded clustering method, which simultaneously learns feature representation and clustering via explicit modeling of scRNA-seq data generation.

**scAce [2]**: scAce is an adaptive embedding and clustering method, which constructs a VAE to simultaneously learn cell embeddings and cluster assignments.

**AttentionAE-sc [3]**: AttentionAE-sc (Attention fusion AutoEncoder for single-cell) is a novel scRNA-seq data clustering method, in which two different scRNA-seq clustering strategies (zero-inflated negative binomial (ZINB)-based and graph autoencoder (GAE)-based methods) are combined.

**scMCKC [4]**: scMCKC is a deep clustering framework for single-cell data which integrates a ZINB-based autoencoder with multiple constraints to improve clustering accuracy. It enhances cluster compactness through similarity-based cell associations and incorporates prior pairwise constraints, while using a weighted soft K-means algorithm to refine cell population assignments.

**scGAC [5]**: scGAC is a novel unsupervised clustering method, which integrates graph attention and autoencoding to learn discriminative, clustering-oriented cell representations. It refines cell graphs through network denoising, propagates information with attention-weighted message passing, and employs a self-optimizing strategy to determine final cell clusters.

**Baseline implementation and comparison settings**

We also included several simple control baselines, including PCA + k-means, PCA/UMAP + Leiden clustering, NMF-based representation + k-means, and standard autoencoder + k-means. All competing methods started from the same log2-transformed single-cell proteomics matrices described in Section Datasets and preprocessing, with missing values replaced by zero. Method-specific preprocessing required by the corresponding implementations was then applied where necessary. For methods originally developed for scRNA-seq count data, we did not reconstruct artificial count data from the proteomics measurements; instead, count-specific normalization or transformation steps were omitted where supported by the implementation.

Unless otherwise specified, baseline model hyperparameters followed the recommended or default settings reported in the corresponding publications or implementations, without selection according to ground-truth ARI or NMI. The only explicit baseline parameter scan was the Leiden resolution search in the known-*K* guided setting, where the resolution was selected according to the annotated cluster number rather than clustering accuracy. Software versions, exact preprocessing procedures, hyperparameter settings and tuning procedures, and cluster-number specifications for all competing methods are summarized in Supplementary Table S8.

For scYOU, the final model settings and predefined dataset-size rules for the reconstruction weight and learning rate are reported in Table 3, while sensitivity analyses of the principal model hyperparameters are provided in Supplementary Note S1, with complete loss-related and latent-dimensionality results reported in Supplementary Tables S9 and S10, respectively.

Notably, all methods were applied to the same protein expression matrices described in Section Datasets and preprocessing, which were log-transformed and normalized using standard single-cell proteomics pipelines, rather than transcriptomics-specific preprocessing procedures. All baseline methods were evaluated using their officially released implementations and default hyperparameter settings.

### Computational recovery of complex-associated protein modules

#### Curated reference protein complex database and protein–protein interaction network construction

Curated reference protein complex collections were constructed by integrating multiple established resources and filtering them according to protein coverage in each proteomics dataset. For human datasets, the reference collection integrates RCSB PDB ([17]), CORUM (Comprehensive Resource of Mammalian protein complexes [18]), Hu.MAP (Human Multi-protein Assembly Prediction [19]), ComplexPortal (EBI [20]), and Reactome Complexes ([21]); for mouse datasets, RCSB PDB, CORUM, ComplexPortal, and BioGRID [22] were used. Complexes whose complete subunit sets were covered by the corresponding proteomics dataset were retained as the dataset-specific reference complex set for computational evaluation. A total of 26984 human complexes and 7433 mouse complexes were finally collected (Supplementary Tables S11). During benchmark integration, exact duplicate complexes with identical protein compositions were removed, whereas partially overlapping or nested complexes with different subunit compositions were retained as independent reference complexes. These entries were preserved because they correspond to distinct curated complex annotations and may represent related but compositionally different biological assemblies. For each single-cell proteomics dataset, the integrated reference collection was further filtered according to protein coverage, and only complexes whose complete subunit sets were contained in the corresponding omics dataset were retained as reference complexes. We note that, because overlapping reference complexes are retained, a predicted module sharing subunits with multiple related complexes may satisfy the matching criterion for more than one reference complex, which can influence the absolute values of benchmark metrics. Nevertheless, the same dataset-specific reference set and evaluation protocol were applied consistently to all compared methods, ensuring a fair relative comparison. Complex-associated module recovery was evaluated only on datasets with sufficient coverage of curated reference complexes. Montalvo was excluded because it contained no fully covered reference complexes, whereas Huffman was excluded because only three fully covered reference complexes were available, which was considered insufficient for a stable complex-recovery evaluation. Accordingly, subsequent module-recovery experiments were conducted on the four human datasets with substantially larger curated reference sets.

We used STRING ([23]), one of the largest and most authoritative PPI network databases. For the complex-associated module recovery task, we constructed PPI networks by extracting the subgraphs induced by proteins in the curated reference complexes under different STRING interaction-score (confidence) thresholds. As shown in Table 2, due to the paucity of mouse complex data, four human datasets were used to evaluate the ability of scYOU-guided PPI refinement to recover curated complex-associated organization. In order to test the robustness of the embedding to complex recognition, we constructed networks with different interaction strengths (0,150,400,700,900) and fed them into the complex recognition algorithm, and used F1 and ACC to evaluate (Section Performance metrics). In order to prevent overfitting, Euclidean- and cosine-distance–based similarity measures (Equation (28)) are employed to quantify protein similarity in the omics matrix.


(28) \begin{align*}& \mathbf{Sim}_{ij} = \rho(\mathbf{Z}_{\textrm{protein}}^{i},\mathbf{Z}_{\textrm{protein}}^{j}),\end{align*}

**Table 2:** Summary statistics of omics-covered complexes and induced STRING PPI networks (interaction score threshold = 400).

Metric	Khan	Specht	Montalvo	Leduc-TMT32	Huffman	Leduc-plexDIA
Complexes (*≥ *1 protein in omics)	12 855	12 943	145	9971	408	15 209
Complexes (all proteins in omics)	158	165	0	273	3	809
Proteins in omics	3178	3042	501	1775	1123	4619
Proteins in STRING	3128	3001	500	1767	1103	4256
Proteins in reference complexes	316	327	0	465	8	1273
PPIs among reference-complex proteins	8060	10 320	0	21 922	12	63 248

Interaction score threshold is fixed to 400 in STRING. Fully covered reference complexes are defined as curated complexes whose complete subunit sets are covered by the corresponding proteomics dataset. The induced PPI count refers to the number of STRING interactions among proteins contained in the fully covered reference complexes under the given threshold.

**Table 3:** Implementation details and hyperparameter settings used in scYOU.

Category	Setting	Details
Network architecture	Cell autoencoder	Encoder: $d_{\textrm{in}}\rightarrow 256\rightarrow 64\rightarrow 32$; decoder: $32\rightarrow 64\rightarrow 256\rightarrow d_{\textrm{in}}$.
	Protein autoencoder	Encoder: $n\rightarrow 256\rightarrow 64\rightarrow 32$; decoder: $32\rightarrow 64\rightarrow 256\rightarrow n$.
	Activation / regularization	ReLU hidden activations; no dropout; no batch normalization.
Latent representation	Embedding dimension	Cell embedding = 32; protein embedding = 32.
Optimization	Optimizer	Adam with AMSGrad; weight decay $1\times 10^{-5}$; gradient clipping norm 1.0.
	Training mode	Full-batch optimization.
	Training epochs	Max 2000 pretraining epochs; max 2000 refinement epochs.
	Early stopping	Threshold $1\times 10^{-4}$; patience 15 epochs.
	Initialization	K-means initialization for cluster centers; default PyTorch initialization otherwise.
Clustering	Cluster number	Known-K benchmark: annotated number of cell populations; label-free evaluation: estimated using the spectral eigengap procedure described in Section Label-free estimation of the cluster number.
	Target update	Updated every 10 epochs.
	Student’s *t* parameter	Degree of freedom *ν =1.0*.
Loss-related parameters	Fixed weights	*β =0.1* ; *γ =1.0*; *δ =1.0*; *τ =0.5*.
	Reconstruction weight	*α =1.0* for *n≤ 1500*; *α =0.5* for *n>1500*.
Learning rate	Dataset-size rule	$1\times 10^{-3}$ for *n≤ 1000*; $5\times 10^{-4}$ for *1000<n≤ 1500*; $1\times 10^{-4}$ for *n>1500*.
Supercell construction	Graph parameters	Top-neighbor parameter *a=5*; shared-neighbor threshold *b=2*.

where $\mathbf{Z}_{\textrm{protein}}^{i}$ denotes the embedding vector of the *i*th protein, and $\rho (\cdot ,\cdot )$ represents a similarity function induced by either Euclidean distance or cosine distance, defined as


\begin{align*} & 1 - \frac{\lVert \mathbf{Z}_{\textrm{protein}}^{i} - \mathbf{Z}_{\textrm{protein}}^{j} \rVert_{2}} {\max_{p,q}\lVert \mathbf{Z}_{\textrm{protein}}^{p} - \mathbf{Z}_{\textrm{protein}}^{q} \rVert_{2}} \quad \textrm{or} \quad \frac{{\mathbf{Z}_{\textrm{protein}}^{i}}^\top \mathbf{Z}_{\textrm{protein}}^{j}} {\lVert \mathbf{Z}_{\textrm{protein}}^{i} \rVert_{2} \lVert \mathbf{Z}_{\textrm{protein}}^{j} \rVert_{2}}. \end{align*}

Based on the calculated similarity between the proteins, the network is reconstructed. Specifically, we complement edges between proteins with high similarity and delete edges between proteins with low similarity. By introducing the original matrix and the matrix information in the embedding space generated by scYOU, we obtained different complex-recovery results.

GO-CC similarity primarily provides information on subcellular localization and associations with CCs or protein-containing complexes, whereas abundance-derived similarity captures co-abundance patterns across cells, while STRING supplies an existing interaction-network context. None of these sources alone constitutes direct evidence that a set of proteins physically assembles into a stable molecular complex. Accordingly, the groups identified in our analysis are referred to as candidate complexes or complex-associated protein modules.

#### Complex-associated module detection and evaluation

To validate the effectiveness of the protein interaction network reconstructed from the scYOU-generated protein embedding matrix, we performed complex-associated module detection on the networks before and after embedding-based reconstruction using two representative complex detection algorithms, ClusterONE and PC2P.

ClusterONE (Clustering with Overlapping Neighborhood Expansion [24]) detects protein complexes by growing cohesive groups from seed proteins through a greedy neighborhood-expansion procedure, and it is designed to identify overlapping complexes, allowing a protein to participate in multiple predicted complexes.

PC2P (Protein Complexes from Coherent Partition [25]) is a parameter-free network-based method that predicts protein complexes by approximating a coherent partition of the PPI network. It employs a greedy approximation strategy grounded in key properties of biclique-spanned subgraphs, enabling complex discovery without manual parameter tuning.

ClusterONE and PC2P are used here as fixed downstream complex-detection algorithms rather than as representation-learning baselines. This design allows us to evaluate whether changes in complex recovery arise from the protein-level representation and PPI refinement rather than from the choice of complex-detection algorithm. The external embedding methods used in the cell-clustering benchmark generate cell-level representations and do not directly output protein embeddings or protein–protein similarity matrices required for this evaluation. We therefore performed the protein-level comparison using STRING-only, GO-only similarity, expression-only similarity, and scYOU embedding-guided PPI refinement under the same downstream detection procedure.

### Label-free estimation of the cluster number

To enable clustering without prior knowledge of the annotated number of cell populations, we adopted a label-free spectral eigengap criterion to estimate the cluster number *K* from the protein expression data. Specifically, the preprocessed cell-by-protein expression matrix was first projected onto a low-dimensional space using principal component analysis (PCA), with up to 32 principal components retained. A symmetric *k*-nearest-neighbor graph was then constructed from the PCA representation, where the neighborhood size was adaptively determined as


(29) \begin{align*}& q=\min\left(30,\max\left(15,\lfloor\sqrt{n}\rfloor\right)\right),\end{align*}

with *n* denoting the number of cells.

Based on the resulting adjacency matrix $\mathbf{A}$, we constructed the normalized graph Laplacian


(30) \begin{align*}& \mathbf{L} = \mathbf{I} - \mathbf{D}^{-1/2} \mathbf{A} \mathbf{D}^{-1/2},\end{align*}

where $\mathbf{D}$ is the corresponding degree matrix.

Let $\lambda _{1}\leq \lambda _{2}\leq \cdots $ denote the ordered eigenvalues of $\mathbf{L}$. Following the spectral eigengap principle [26], the cluster number was estimated as


(31) \begin{align*}& \hat{K} = \mathop{\arg\max}_{K\in\{2,\ldots,10\}} \left(\lambda_{K+1}-\lambda_{K}\right).\end{align*}

The entire estimation procedure is independent of scYOU clustering results and does not use cell-type annotations, ARI, NMI, or other ground-truth information.

### Implementation details and hyperparameter configuration

To improve reproducibility, we summarize the main implementation details and hyperparameter settings of scYOU in Table 3.

### Performance metrics

To evaluate clustering performance, we employed two standard external validation metrics: adjusted rand index (ARI) and normalized mutual information (NMI). Let $\mathcal{T} = \{\mathcal{T}_{1}, \dots , \mathcal{T}_{t}\}$ and $\mathcal{C} = \{\mathcal{C}_{1}, \dots , \mathcal{C}_{p}\}$ represent the ground-truth and predicted partitions, respectively.

ARI measures the pairwise agreement between $\mathcal{T}$ and $\mathcal{C}$, corrected for chance:


(32) \begin{align*}& \textrm{ARI} = \frac{\sum_{ij} \binom{n_{ij}}{2} - \left[\sum_{i} \binom{a_{i}}{2} \sum_{j} \binom{b_{j}}{2}\right] / \binom{n}{2}}{\frac{1}{2}\left[\sum_{i} \binom{a_{i}}{2} + \sum_{j} \binom{b_{j}}{2}\right] - \left[\sum_{i} \binom{a_{i}}{2} \sum_{j} \binom{b_{j}}{2}\right] / \binom{n}{2}},\end{align*}

where $n_{ij} = |\mathcal{T}_{i} \cap \mathcal{C}_{j}|$, $a_{i} = |\mathcal{T}_{i}|$, and $b_{j} = |\mathcal{C}_{j}|$. ARI ranges from *-1* to *1*, where *1* denotes a perfect match and *0* indicates random labeling.

NMI assesses shared information between partitions from an information-theoretic perspective:


(33) \begin{align*}& \textrm{NMI} = \frac{2 \sum_{ij} n_{ij} \log \frac{n n_{ij}}{a_{i} b_{j}}}{-\sum_{i} a_{i} \log \frac{a_{i}}{n} - \sum_{j} b_{j} \log \frac{b_{j}}{n}}.\end{align*}

NMI ranges from *0* to *1*, with higher values indicating stronger consistency between the predicted clusters and the ground truth.

To comprehensively evaluate complex-recovery performance, we followed the evaluation protocol of AdaPPI [27] and reported *Precision*, *Recall*, *ACC*, and *F1* as the main metrics. The corresponding definitions are provided in Equations (34)–(40).

We first compute the positive predictive value (PPV) as


(34) \begin{align*}& \textrm{PPV} = \frac{\sum_{j=1}^{|P_{c}|} \max_{i=1}^{|B_{c}|} T_{ij}}{\sum_{j=1}^{|P_{c}|} N_{j}},\end{align*}

where $P_{c}$ and $B_{c}$ denote the predicted and reference complex sets, respectively. Here, $N_{j}$ is the size of predicted complex *j*, and $T_{ij}$ counts the proteins shared by predicted complex *j* and reference complex *i*.

Following prior work, we define the accuracy index *ACC* by combining PPV with sensitivity (*Sn*):


(35) \begin{align*}& ACC = \sqrt{Sn \cdot PPV},\end{align*}

where sensitivity is calculated as


(36) \begin{align*}& Sn = \frac{\sum_{i=1}^{|B_{c}|} \max_{j=1}^{|P_{c}|} T_{ij}}{\sum_{i=1}^{|B_{c}|} M_{i}},\end{align*}

and $M_{i}$ is the number of proteins in reference complex *i*.

In addition, we assessed prediction quality using pairwise matching under the normalized association (NA) criterion. Specifically, we define


37 \begin{align*} & Precision = \frac{|{p \in P_{c} \mid \exists b \in B_{c} \text{ s.t.}\ NA(p,b) \ge \rho}|}{|P_{c}|},\end{align*}

38 \begin{align*} & Recall = \frac{|{b \in B_{c} \mid \exists p \in P_{c} \text{ s.t.}\ NA(p,b) \ge \rho}|}{|B_{c}|},\end{align*}

39 \begin{align*} & F1 = \frac{2\times Precision\times Recall}{Precision + Recall}, \end{align*}

where the threshold *ρ * is fixed to *0.25* as in Hu *et al.* [28]. The normalized association between a predicted complex *p* and a reference complex *b* is defined as


(40) \begin{align*}& NA(p,b) = \frac{|V_{p} \cap V_{b}|^{2}}{|V_{p}|\cdot|V_{b}|},\end{align*}

with $V_{p}$ and $V_{b}$ denoting the subunit (protein) sets of complexes *p* and *b*, respectively.

## Results

### Overview of scYOU

In this section, we present an overview of scYOU (Fig. 2), a multi-scale Yoked Optimization and Unsupervised framework that jointly models cellular heterogeneity and protein-level functional organization from proteomics data. scYOU adopts a dual-channel architecture that learns cell embeddings and protein embeddings simultaneously, and introduces a supercell construction strategy that adaptively combines identity-level correspondence for singleton cells with neighborhood-level cross-cell constraints for locally supported cell groups. To couple the two views, scYOU further employs a dual-channel alignment objective that pulls cells within the same supercell closer while separating cells from different supercells, encouraging consistent representations across modeling perspectives. On the protein side, scYOU injects GO-based functional similarity priors to regularize protein representations, enabling the learned embeddings to support downstream interaction refinement and recovery of complex-associated protein modules. We then benchmark scYOU across cell clustering performance and visualization quality, assess biological interpretability via differential protein expression analysis, demonstrate that protein embeddings improve recovery of curated complex-associated organization through embedding-guided PPI pruning, and finally validate key design choices through ablation studies on supercell construction and alignment.

**Figure 2:**
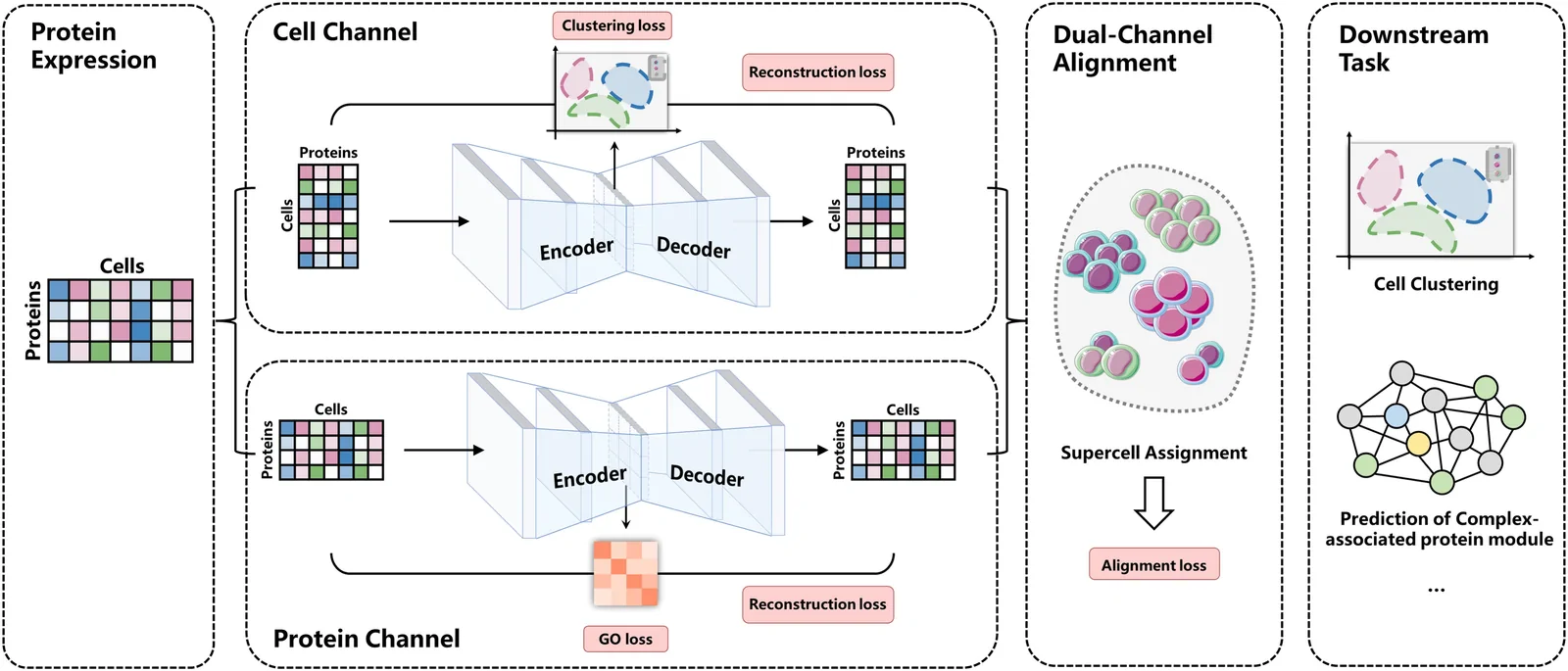
Overview of the scYOU framework, illustrating parallel cell and protein autoencoders, supercell-guided alignment of reconstructed expression profiles with identity-level and neighborhood-level correspondence, channel-specific clustering and GO-based refinement objectives, and downstream applications to cellular heterogeneity analysis and complex-associated protein module recovery.

### scYOU maintains strong clustering performance under both known- and estimated-*K* settings

We evaluated scYOU and competing methods across six single-cell proteomics datasets using ARI and NMI. To explicitly distinguish clustering quality from estimation of the number of cell populations, we considered two complementary evaluation settings. In the controlled known-*K* benchmark, methods requiring a predefined cluster number were supplied with the annotated number of cell populations; for Leiden-based methods, the resolution was guided solely by the annotated cluster number. In the label-free evaluation, scYOU estimated *K* directly from the expression data without using cell-type annotations, whereas competing methods were evaluated using their original or default label-free clustering procedures where applicable. All methods were evaluated using ten random seeds. Paired comparisons across matched seeds were performed using paired *t*-tests with Benjamini–Hochberg FDR correction, with paired Wilcoxon signed-rank tests as a non-parametric sensitivity analysis. Complete statistical results are provided in Supplementary Table S12.

**Controlled known-*K* benchmark**

Under the controlled known-*K* setting, scYOU achieved the highest mean ARI (Table 4) and NMI (Table 5) on five of the six datasets. Relative to the strongest competing method on each dataset, paired comparisons supported significant improvements after FDR correction on Montalvo, Specht, Huffman, and Leduc-TMT32 for both metrics. On Khan, scYOU retained the highest mean ARI and NMI, although the paired differences were not statistically significant, indicating comparable performance among the leading methods. On Leduc-plexDIA, several methods approached the performance ceiling: scYOU differed from the best ARI by only 0.0024 without a significant difference, while the best NMI exceeded scYOU by only 0.0030.

**Table 4:** Cell clustering performance under the controlled known-*K* setting in terms of **ARI**.

${}_{\textrm{Methods}}\Big\backslash\! {}^{\textrm{Datasets}}$	Montalvo	Specht	Khan	Huffman	Leduc-plexDIA	Leduc-TMT32
scPROTEIN	0.6972*± *0.0396	0.3205*± *0.0456	0.1787*± *0.0450	0.9000*± *0.0367	0.9730*± *0.0232	0.7553*± *0.0437
scPGCL	0.2579*± *0.0192	0.2008*± *0.0071	0.1328*± *0.0102	0.2922*± *0.0293	0.7700*± *0.0250	0.3860*± *0.0478
omicsGMF	0.3242*± *0.0640	0.3103*± *0.0308	0.2127*± *0.0177	0.2423*± *0.0345	0.8660*± *0.0834	0.6875*± *0.1964
scDeepCluster	0.4877*± *0.0451	0.2733*± *0.0345	0.2076*± *0.0194	0.6732*± *0.0495	0.9798*± *0.0168	0.3863*± *0.0488
scAce	0.4352*± *0.0447	0.2931*± *0.0443	0.1975*± *0.0316	0.6571*± *0.0591	0.8972*± *0.0541	0.4856*± *0.0436
AttentionAE-sc	0.5188*± *0.0500	0.2851*± *0.0476	0.1800*± *0.0306	0.5824*± *0.0578	0.9744*± *0.0434	0.6459*± *0.0476
scMCKC	0.5552*± *0.0593	0.2899*± *0.0323	0.2223*± *0.0300	0.8418*± *0.0299	0.9820*± *0.0171	0.6488*± *0.0293
scGAC	0.5312*± *0.0542	0.3135*± *0.0591	0.1682*± *0.0415	0.8973*± *0.0362	0.9418*± *0.0494	0.5764*± *0.0383
PCA-KMeans	0.2776*± *0.0143	0.2943*± *0.0141	0.1769*± *0.0177	0.4520*± *0.0373	0.9748*± *0.0303	0.5567*± *0.0297
NMF-KMeans	0.2356*± *0.1074	-0.0505*± *0.0709	-0.0116*± *0.0252	0.2191*± *0.2077	0.0264*± *0.0305	-0.0002*± *0.0223
AE-KMeans	0.3018*± *0.0224	0.2746*± *0.0627	0.1556*± *0.0283	0.9073*± *0.0266	0.8934*± *0.0741	0.0154*± *0.0310
PCA-Leiden (known-*K* guided)	0.4690*± *0.0620	0.2080*± *0.1355	0.1486*± *0.0169	0.9153*± *0.0259	0.9918*± *0.0085	0.5569*± *0.0116
UMAP-Leiden (known-*K* guided)	0.2679*± *0.0334	0.1438*± *0.0416	0.1983*± *0.0370	0.8449*± *0.0297	**0.9922*± *0.0075**	0.2543*± *0.0369
scYOU	**0.8153*± *0.0219**	**0.3603*± *0.0418**	**0.2389*± *0.0075**	**0.9366*± *0.0110**	0.9898*± *0.0132	**0.8856*± *0.0279**

Methods requiring a predefined cluster number were supplied with the annotated number of cell populations. Results are reported as mean *± * standard deviation over ten random seeds. The best result is highlighted in bold and the second-best result is underlined.

**Table 5:** Cell clustering performance under the controlled known-*K* setting in terms of **NMI**.

${}_{\textrm{Methods}}\Big\backslash\! {}^{\textrm{Datasets}}$	Montalvo	Specht	Khan	Huffman	Leduc-plexDIA	Leduc-TMT32
scPROTEIN	0.7059*± *0.0271	0.3770*± *0.0338	0.3012*± *0.0287	0.8451*± *0.0379	0.9642*± *0.0367	0.7629*± *0.0436
scPGCL	0.5465*± *0.0265	0.1994*± *0.0088	0.2396*± *0.0063	0.2911*± *0.0372	0.7790*± *0.0270	0.5596*± *0.0467
omicsGMF	0.5934*± *0.0511	0.3729*± *0.0138	0.3041*± *0.0158	0.1847*± *0.0285	0.8568*± *0.0842	0.7336*± *0.1419
scDeepCluster	0.6547*± *0.0452	0.3553*± *0.0343	0.2949*± *0.0229	0.7048*± *0.0555	0.9791*± *0.0324	0.6371*± *0.0469
scAce	0.6218*± *0.0567	0.3755*± *0.0287	0.3270*± *0.0246	0.6979*± *0.0595	0.9088*± *0.0634	0.6248*± *0.0422
AttentionAE-sc	0.6589*± *0.0449	0.3601*± *0.0550	0.2708*± *0.0270	0.6236*± *0.0561	0.9705*± *0.0263	0.7018*± *0.0428
scMCKC	0.6520*± *0.0508	0.3664*± *0.0329	0.3079*± *0.0257	0.7958*± *0.0518	0.9752*± *0.0250	0.5604*± *0.0552
scGAC	0.6328*± *0.0370	0.3530*± *0.0366	0.2886*± *0.0315	0.8325*± *0.0271	0.9487*± *0.0375	0.6534*± *0.0600
PCA-KMeans	0.5730*± *0.0249	0.3652*± *0.0323	0.2964*± *0.0321	0.3767*± *0.0155	0.9584*± *0.0246	0.6339*± *0.0208
NMF-KMeans	0.3724*± *0.0812	0.0777*± *0.0257	0.1155*± *0.0407	0.2779*± *0.1810	0.1167*± *0.0608	0.0081*± *0.0131
AE-KMeans	0.5691*± *0.0302	0.3585*± *0.0342	0.2541*± *0.0368	0.8648*± *0.0409	0.9048*± *0.0779	0.0335*± *0.0299
PCA-Leiden (known-*K* guided)	0.6564*± *0.0411	0.2181*± *0.1058	0.2474*± *0.0224	0.8663*± *0.0307	**0.9907*± *0.0078**	0.5074*± *0.0132
UMAP-Leiden (known-*K* guided)	0.5489*± *0.0516	0.2677*± *0.0433	0.2947*± *0.0196	0.7948*± *0.0306	0.9852*± *0.0150	0.3941*± *0.0313
scYOU	**0.7517*± *0.0198**	**0.4003*± *0.0243**	**0.3333*± *0.0044**	**0.8858*± *0.0149**	0.9877*± *0.0098	**0.8625*± *0.0248**

Experimental settings and highlighting follow Table 4.

Importantly, scYOU also showed a consistent advantage over proteomics-specific representation methods. Across all six datasets, scYOU achieved higher ARI and NMI than scPROTEIN, scPGCL, and omicsGMF, with all corresponding paired comparisons remaining significant after FDR correction. These results provide further evidence for the competitive performance of scYOU relative to methods specifically developed for proteomics data.

The two smallest datasets, Huffman and Leduc-plexDIA, were interpreted more cautiously because of their limited sample sizes and relatively strong baseline performance, particularly on Leduc-plexDIA where several methods were already close to perfect clustering. We therefore place greater emphasis on the larger and more heterogeneous datasets when assessing comparative clustering performance.

**Label-free estimated-*K* benchmark**

We next evaluated clustering without access to the annotated number of cell populations. For scYOU, the spectral eigengap procedure described in Section Label-free estimation of the cluster number estimated *\hat K=3,2,7,2,3,* and *6* for Montalvo, Specht, Khan, Huffman, Leduc-plexDIA, and Leduc-TMT32, respectively, compared with annotated values of *5,2,6,2,3,* and *3*. Competing methods were evaluated using their original or default cluster-number determination procedures without access to the annotated cluster count. PCA-Leiden and UMAP-Leiden were evaluated in their unguided forms without annotated-*K*-based resolution selection.

Under the label-free setting, scYOU achieved the highest mean ARI (Table 6) and NMI (Table 7) on all six datasets. Its ARI improvements over the strongest competing method were statistically significant after FDR correction on all six datasets, while the NMI improvements were significant on five datasets. On Khan, scYOU retained the highest mean NMI (*0.3400± 0.0087* versus *0.3235± 0.0364* for scAce), although the paired difference was not statistically significant. Strong performance was also retained when the data-derived *\hat K* differed from the annotation-defined cluster number, supporting the applicability of scYOU when the number of cell populations is unavailable.

**Table 6:** Cell clustering performance under the label-free estimated-*K* setting in terms of **ARI**.

${}_{\textrm{Methods}}\Big\backslash\! {}^{\textrm{Datasets}}$	Montalvo	Specht	Khan	Huffman	Leduc-plexDIA	Leduc-TMT32
scDeepCluster	0.4346*± *0.0585	0.2255*± *0.0397	0.1603*± *0.0284	0.6166*± *0.0773	0.9707*± *0.0220	0.3340*± *0.0537
scAce	0.3824*± *0.0707	0.2432*± *0.0544	0.1569*± *0.0453	0.6197*± *0.0639	0.8548*± *0.0704	0.4371*± *0.0407
AttentionAE-sc	0.4622*± *0.0460	0.2313*± *0.0617	0.1808*± *0.0318	0.5211*± *0.0751	0.9364*± *0.0299	0.6547*± *0.0324
PCA-Leiden (unguided)	0.1629*± *0.0375	0.0807*± *0.0251	0.1513*± *0.0348	0.4288*± *0.0089	0.9157*± *0.0440	0.1929*± *0.0124
UMAP-Leiden (unguided)	0.0679*± *0.0198	0.0456*± *0.0221	0.0908*± *0.0344	0.1746*± *0.0354	0.5024*± *0.0389	0.1079*± *0.0196
scYOU	**0.8584*± *0.0233**	**0.3603*± *0.0418**	**0.2382*± *0.0120**	**0.9366*± *0.0110**	**0.9898*± *0.0132**	**0.7985*± *0.0323**

No annotated cluster-number information was used. Results are reported as mean *± * standard deviation over ten random seeds. The best result is highlighted in bold and the second-best result is underlined.

**Table 7:** Cell clustering performance under the label-free estimated-*K* setting in terms of **NMI**.

${}_{\textrm{Methods}}\Big\backslash\! {}^{\textrm{Datasets}}$	Montalvo	Specht	Khan	Huffman	Leduc-plexDIA	Leduc-TMT32
scDeepCluster	0.6115*± *0.0571	0.2999*± *0.0603	0.3000*± *0.0259	0.6549*± *0.0524	0.9735*± *0.0201	0.5935*± *0.0410
scAce	0.5665*± *0.0337	0.3167*± *0.0554	0.3235*± *0.0364	0.6430*± *0.0510	0.8586*± *0.0656	0.5723*± *0.0518
AttentionAE-sc	0.6031*± *0.0565	0.3198*± *0.0513	0.2769*± *0.0410	0.5657*± *0.0650	0.9272*± *0.0445	0.7067*± *0.0360
PCA-Leiden (unguided)	0.4767*± *0.0274	0.2197*± *0.0298	0.3152*± *0.0241	0.5722*± *0.0142	0.9047*± *0.0163	0.3986*± *0.0263
UMAP-Leiden (unguided)	0.3898*± *0.0312	0.2257*± *0.0239	0.3041*± *0.0287	0.4081*± *0.0218	0.7211*± *0.0188	0.3555*± *0.0454
scYOU	**0.7549*± *0.0235**	**0.4003*± *0.0243**	**0.3400*± *0.0087**	**0.8858*± *0.0149**	**0.9877*± *0.0098**	**0.7965*± *0.0350**

Experimental settings and highlighting follow Table 6.

**Sensitivity to the specified cluster number**

To further assess sensitivity to cluster-number misspecification, we varied the input *K* from 1 to 10 while keeping the remaining model settings fixed (Fig. 3). Clustering performance was generally highest at or near the annotation-defined *K*, while the degree of sensitivity varied across datasets. Montalvo and Khan maintained relatively stable performance over several neighboring values of *K*, whereas Specht and Leduc-plexDIA showed a clearer dependence on cluster-number specification. Huffman and Leduc-TMT32 retained relatively high performance under moderate misspecification but showed larger decreases as *K* moved further from the annotation-defined value. Thus, scYOU can tolerate moderate cluster-number misspecification on several datasets, although the effect of *K* remains dependent on the underlying data structure.

**Figure 3:**
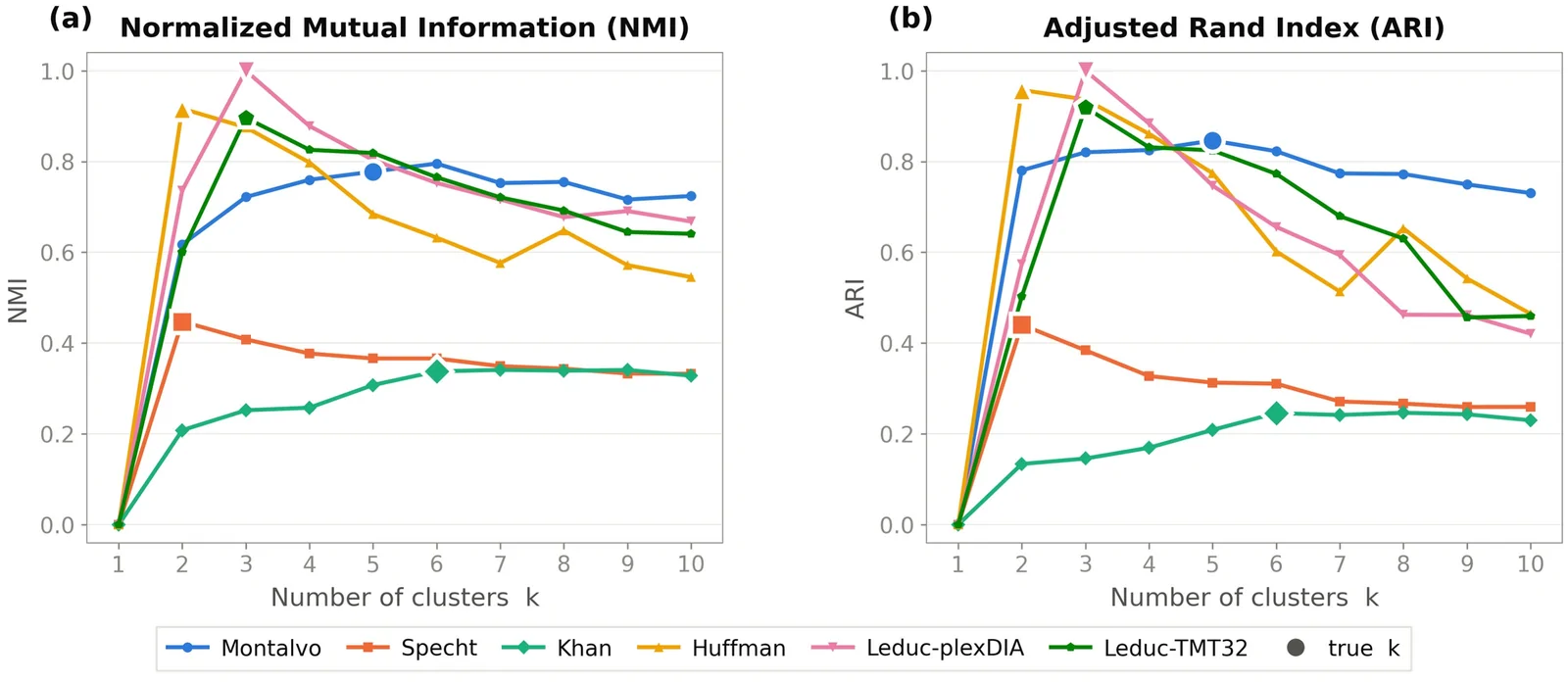
Sensitivity of scYOU to the specified number of clusters. (a) NMI and (b) ARI obtained by varying the input cluster number *K* from 1 to 10 across the six datasets while keeping the remaining model settings fixed. Enlarged markers indicate the annotation-defined cluster number for each dataset. Clustering performance generally peaks at or near the annotation-defined *K*, while the degree of sensitivity to cluster-number misspecification varies across datasets.

Taken together, the controlled and label-free evaluations show that scYOU remains competitive across different clustering settings. The stronger evidence for comparative performance comes from the larger and more heterogeneous datasets, whereas the two smallest datasets are interpreted more cautiously. Importantly, expanding the proteomics-specific baseline pool did not alter the overall ranking, and removal of the annotated cluster count likewise did not eliminate the performance advantage of scYOU.

### t-SNE visualization illustrates cell-type structure in learned representations

To qualitatively assess the separability of the learned cell representations, we visualized the low-dimensional embeddings using t-distributed stochastic neighbor embedding (t-SNE). For each representation-learning method, t-SNE was applied to the corresponding learned cell representation, while the processed protein-expression matrix was used directly for the raw-expression reference. Cells were colored according to their annotated cell types, and the same visualization procedure was used across methods.

Figure 4 shows a representative comparison on the Specht dataset, including the raw expression space, proteomics-oriented methods, transcriptomics-derived clustering methods, and scYOU. In the scYOU projection, the two annotated cell populations exhibit a clearer class-associated organization than in the raw expression space, with cells forming two major regions connected by a relatively limited transition area. Although overlap remains between the two populations, consistent with the moderate ARI and NMI values on this challenging dataset, scYOU retains a readily recognizable group-level organization. Several competing representations also exhibit partial class separation, whereas others show stronger mixing or fragmentation into multiple disconnected regions in the two-dimensional projection.

**Figure 4:**
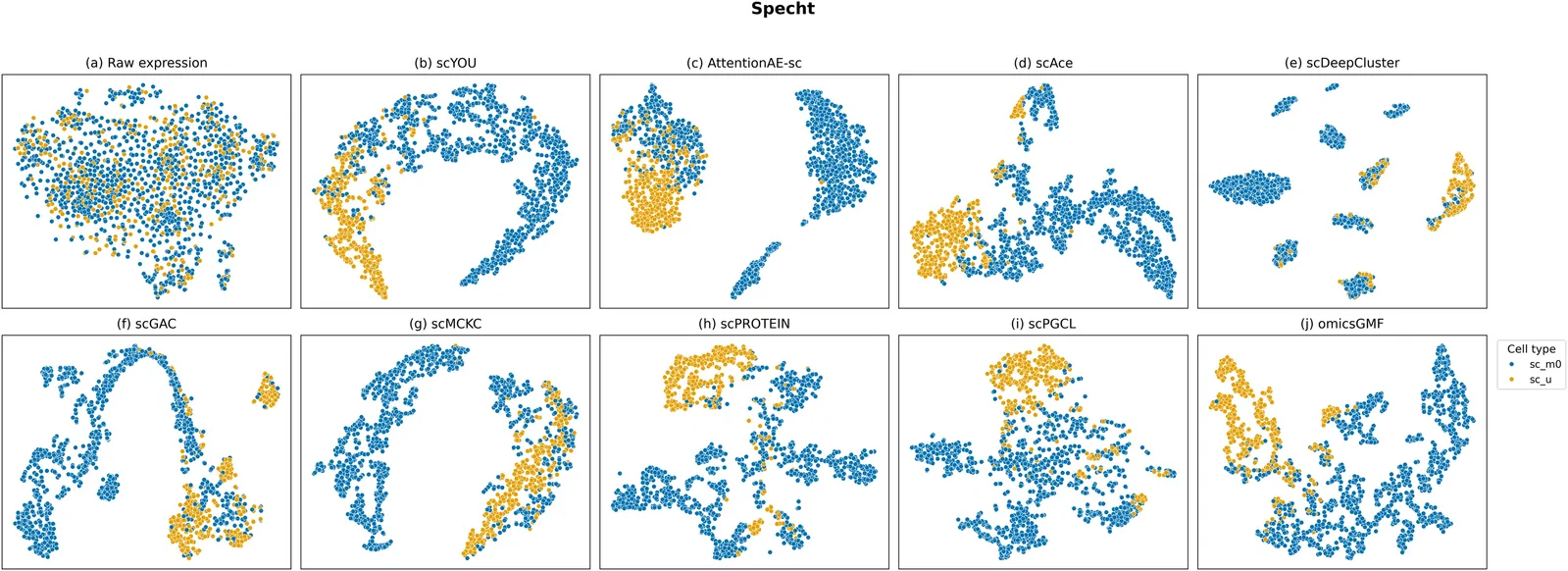
t-SNE visualization of cell representations on the Specht dataset, comparing processed expression features with embeddings obtained from competing methods and scYOU, with points colored according to annotated cell types.

The complete t-SNE comparisons for the remaining five datasets are provided in Supplementary Figs S3–S7. On Montalvo and Huffman, scYOU produces compact regions with clear cell-type-associated organization. Leduc-plexDIA is already highly separable in the processed expression space, and most methods retain the three clearly distinguishable cell populations, consistent with the near-ceiling clustering performance observed on this dataset. On Khan, substantial class overlap remains across the different representations, reflecting the greater difficulty of separating the six annotated populations, although scYOU maintains recognizable local group structure. On Leduc-TMT32, scYOU separates the three annotated populations into well-organized regions, while several other representation-learning methods also recover substantial cell-type structure to varying degrees. Overall, these qualitative patterns are broadly consistent with the quantitative ARI and NMI results and indicate that the clustering performance of scYOU is accompanied by a cell representation in which annotation-associated structure remains visually discernible across datasets of different difficulty.

### Biological interpretation of scYOU-derived clusters on the Leduc-TMT32 dataset

To assess the biological interpretability of scYOU-derived clusters on the Leduc-TMT32 dataset, we performed cluster-level differential protein analysis followed by GO enrichment analysis, including BP, MF, and CC terms. For each cluster, cluster-enriched proteins were identified by comparing cells in the target cluster against all remaining cells using a one-sided Welch’s t-test with Benjamini–Hochberg FDR correction. We also applied the same differential protein analysis and GO enrichment pipeline to representative baseline-derived clusters from scPROTEIN, AttentionAE-sc, scGAC, and PCA-Leiden, covering single-cell proteomics-specific, deep autoencoder-based, graph-based, and conventional clustering strategies, respectively. For all methods, GO over-representation analysis was performed using the same experimental protein background and propagated GO annotations, with BP, MF, and CC terms tested separately using one-sided hypergeometric tests followed by Benjamini–Hochberg FDR correction within each ontology. The complete marker lists are provided in Supplementary Tables S13A–S17B, while the complete cross-method GO enrichment results are provided in Supplementary Table S18. Representative GO enrichment patterns derived from scYOU are shown in Fig. 5.

**Figure 5:**
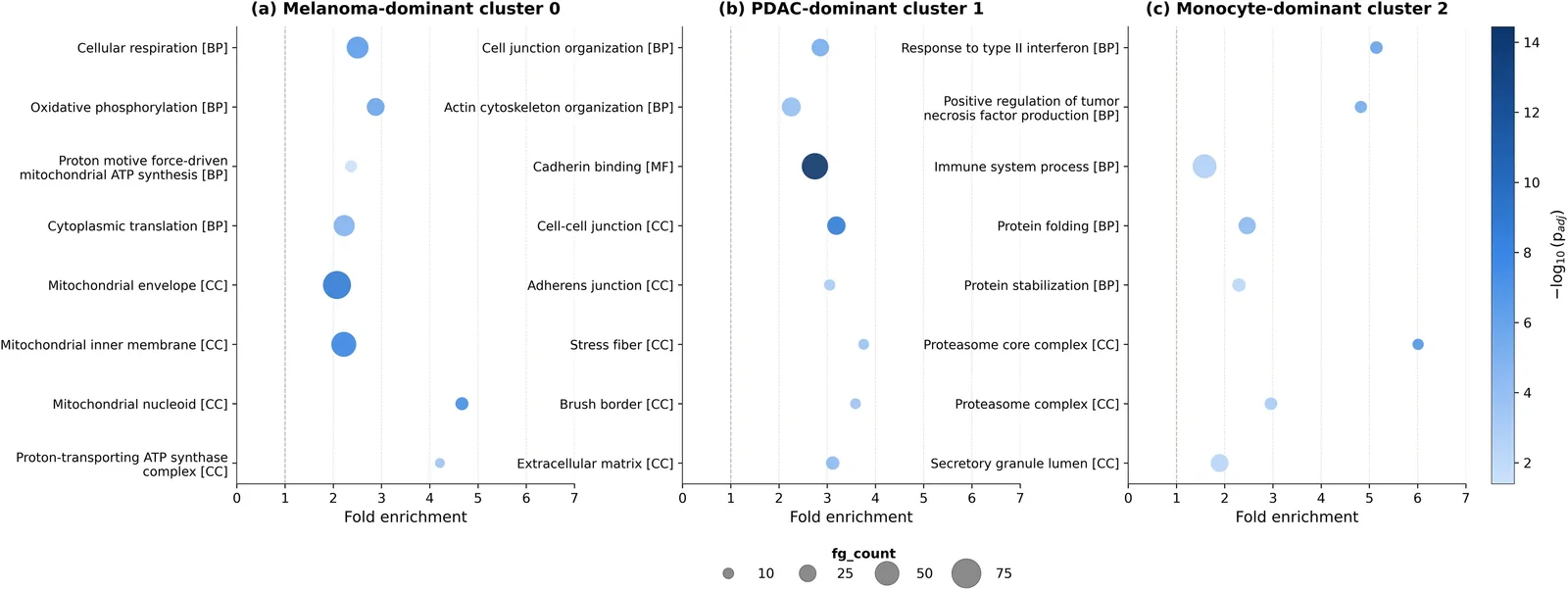
GO enrichment analysis of scYOU-derived cluster-enriched proteins on the Leduc-TMT32 dataset. (a–c) Bubble plots showing representative significantly enriched GO terms for cluster 0 (melanoma-dominant), cluster 1 (PDAC-dominant), and cluster 2 (monocyte-dominant), respectively. The x-axis represents fold enrichment, bubble size indicates the number of cluster-enriched proteins associated with each GO term, and color denotes the $-\log _{10}(\textrm{FDR})$. GO terms from BP, MF, and CC are indicated in the term labels.

Cluster 0 was dominated by melanoma cells and contained 280 significant marker proteins, showing a melanoma-associated protein program. Representative markers included VIM/P08670, RAB7A/P51149, LGALS3/P17931, SHMT2/P34897, and HSPD1/P10809. These markers were highly ranked by effect size in scYOU, with SHMT2 ranked 1st, VIM 5th, RAB7A 10th, HSPD1 17th, and LGALS3 18th; all showed strong enrichment effects, with Cohen’s *d* ranging from 1.33 to 2.20 and FDR-adjusted *q*-values from $10^{-84}$ to $10^{-176}$. VIM has been associated with melanoma diagnosis and metastatic potential [29], RAB7A regulates melanosome maturation through gp100 processing [30, 31], and LGALS3 contributes to melanoma growth and metastasis [32]. Consistently, GO enrichment highlighted “cellular respiration,” “oxidative phosphorylation,” “proton motive force-driven mitochondrial ATP synthesis,” “mitochondrial inner membrane,” “mitochondrial nucleoid,” “proton-transporting ATP synthase complex,” and “cytoplasmic translation” (Fig. 5a), indicating a melanoma-associated metabolic state characterized by mitochondrial energy production and active protein synthesis [33]. In comparison, scPROTEIN recovered a similar melanoma marker profile, with the same five markers ranked within the top 25 in its melanoma-dominant cluster. However, the melanoma-associated markers were less consistently ranked in other baselines: scGAC did not identify LGALS3 as a significant marker and ranked VIM and RAB7A only 87th and 90th, respectively; AttentionAE-sc and PCA-Leiden split melanoma-dominant cells into multiple clusters, where several markers were either absent or ranked much lower, such as HSPD1 ranked 195th in one AttentionAE-sc melanoma cluster and 180th in one PCA-Leiden melanoma cluster. Cross-method GO enrichment analysis showed that the core mitochondrial program was also recovered by the matched melanoma-dominant baseline clusters, including “cellular respiration,” “oxidative phosphorylation,” “proton motive force-driven mitochondrial ATP synthesis,” and “mitochondrial nucleoid.” Among the representative terms highlighted in Fig. 5a, scYOU additionally retained significant enrichment of cytoplasmic translation, whereas this term did not reach the FDR threshold in the corresponding baseline clusters (Supplementary Table S18).

Cluster 1 corresponded to PDAC cells and contained 269 significant marker proteins, displaying a PDAC-like epithelial and invasive program. Representative markers included KRT18/P05783, ANXA2/P07355, FSCN1/Q16658, EZR/P15311, and MYH9/P35579. These proteins were among the most strongly enriched markers in scYOU, ranking from 5th to 17th by effect size, with Cohen’s *d* values from 2.55 to 3.27 and extremely significant *q*-values, including several approaching zero after FDR correction. KRT18 supports epithelial identity [34], ANXA2 has been linked to PDAC progression and poor prognosis [35], and FSCN1 promotes actin remodeling, invasion, and metastasis in PDAC [36]. GO enrichment further identified “cell junction organization,” “actin cytoskeleton organization,” “stress fiber,” “adherens junction,” “cell-cell junction,” “cadherin binding,” “brush border,” and “extracellular matrix” (Fig. 5b). These terms are consistent with epithelial adhesion, cytoskeletal remodeling, and extracellular-matrix interaction in PDAC [36, 37]. The baseline comparison showed that PDAC-related markers were also strongly recovered by scPROTEIN and scGAC, with the five representative markers ranked within the top 18 and showing comparable effect sizes. In contrast, PCA-Leiden recovered the same PDAC markers but with weaker effect sizes, with Cohen’s *d* values reduced to *∼ *1.75–2.10. AttentionAE-sc also detected these markers in its major PDAC-dominant cluster, but PDAC cells were split into multiple clusters, including very small clusters in which only part of the marker set could be detected. Consistent with the marker-level results, the matched PDAC-dominant clusters from all representative baseline methods also recovered the major adhesion- and cytoskeleton-related GO terms, including “actin cytoskeleton organization,” “cell-cell junction,” “cadherin binding,” “stress fiber,” and “extracellular matrix” (Supplementary Table S18). These results suggest that the PDAC program was a strong signal in this dataset, while scYOU recovered it within a single compact and highly coherent cluster.

Cluster 2 corresponded to monocytes and contained 239 significant marker proteins, supporting a monocyte/myeloid immune program. Representative markers included LCP1/P13796, RAC2/P15153, CORO1A/P31146, and MSN/P26038, which are related to leukocyte cytoskeletal regulation and phagocyte function [38, 39]. In scYOU, LCP1 was ranked 2nd with a Cohen’s *d* of 4.40, while RAC2, MSN, and CORO1A were ranked 18th, 26th, and 38th, respectively, all with significant enrichment after FDR correction. GO enrichment further supported this interpretation, with significant terms including “response to type II interferon,” “positive regulation of tumor necrosis factor production,” “immune system process,” “secretory granule lumen,” “proteasome core complex,” “proteasome complex,” “protein folding,” and “protein stabilization” (Fig. 5c). These results support a monocyte/myeloid state characterized by immune activation, granule-related secretion, and coordinated protein homeostasis. scPROTEIN recovered a similar monocyte marker profile, although with generally lower effect sizes for the same markers. AttentionAE-sc also identified these markers, but distributed them across multiple monocyte-dominant clusters. In scGAC, the monocyte-associated cluster showed weaker recovery of RAC2 and CORO1A, which were ranked 223rd and 191st with much smaller Cohen’s *d* values of 0.35 and 0.41, respectively. PCA-Leiden detected the full marker set in its major monocyte cluster, but several additional monocyte-dominant small clusters lacked most of these representative markers. Thus, compared with these baselines, scYOU provided a more concentrated monocyte/myeloid marker profile within a single cluster. The cross-method GO comparison showed that the major monocyte-associated immune and protein-homeostasis programs were also recovered by scPROTEIN, AttentionAE-sc, and PCA-Leiden, whereas scGAC recovered a somewhat narrower subset of the representative terms; for example, “proteasome complex” and “immune system process” did not reach the FDR threshold in its matched monocyte-dominant cluster (Supplementary Table S18).

Overall, scYOU recovered coherent melanoma-associated, PDAC-like epithelial/invasive, and monocyte/myeloid proteomic programs from a compact and label-consistent clustering structure. The agreement among cluster-enriched proteins, GO-enriched functional programs, and reference annotations supports the biological interpretability of the scYOU-derived clusters. Cross-method enrichment analysis further showed that the major cell-type-associated programs were reproducibly detected across representative baselines, while scYOU retained these signals within a compact three-cluster structure closely aligned with the annotated melanoma, PDAC, and monocyte populations.

### Protein channel embeddings of scYOU facilitate the computational recovery of curated protein complexes

We next evaluated whether scYOU-derived protein embeddings improve the computational recovery of previously curated protein complexes. The reference complex collections were used only for downstream evaluation and were not incorporated into model training. Accordingly, this analysis assesses whether embedding-guided PPI refinement better recovers established complex organization rather than whether scYOU discovers previously unknown physical complexes or novel complex subunits.

To assess the utility of scYOU-derived protein embeddings for curated complex recovery, we used the STRING PPI network as a benchmark and constructed an embedding-guided, pruned interaction network. We then compared complex-recovery performance between the pruned network and the original STRING network alone. As shown in Tables 8 and 9, incorporating scYOU embedding–guided similarity pruning consistently improves ClusterONE performance across all four datasets and across a wide range of STRING interaction score thresholds. At the STRING score threshold of 150, embedding-guided pruning increases the F1-score from 0.0238 to 0.0447 on Specht, from 0.0247 to 0.1480 on Khan, from 0.0000 to 0.0349 on Leduc-TMT32, and from 0.0097 to 0.0271 on Leduc-plexDIA, corresponding to absolute gains of 0.0209, 0.1233, 0.0349, and 0.0174, respectively. As the STRING confidence threshold increases, the absolute F1 scores increase substantially, while embedding-guided pruning continues to improve complex recovery across most dataset-threshold combinations. Notably, the only exception occurs at the strictest threshold (900) on Leduc-TMT32, where F1-score slightly decreases after pruning (0.6102 to 0.6005), suggesting that when the network is already highly stringent, aggressive pruning may remove a small fraction of informative edges.

**Table 8:** F1-score of the complex-detection method ClusterONE on STRING-derived PPI networks filtered by different interaction score thresholds (edges whose STRING interaction scores exceed the threshold are retained).

Score dataset	Specht	Khan	Leduc-TMT32	Leduc-plexDIA
150	0.0238/**0.0447**	0.0247/**0.1480**	0.0000/**0.0349**	0.0097/**0.0271**
400	0.1238/**0.2419**	0.1333/**0.2083**	0.0656/**0.1089**	0.1218/**0.1704**
700	0.4541/**0.5776**	0.5530/**0.7215**	0.3043/**0.5506**	0.2967/**0.3927**
900	0.6956/**0.7154**	0.7510/**0.7846**	**0.6102**/0.6005	0.5571/**0.6248**

Values are reported as “without scYOU / with scYOU,” where “without scYOU” denotes curated complex recovery using the original STRING PPI network, and “with scYOU” denotes curated complex recovery after scYOU embedding-guided PPI refinement. Bold values indicate results obtained with scYOU embedding-guided PPI refinement.

**Table 9:** Accuracy of the complex-detection method ClusterONE on STRING-derived PPI networks filtered by different interaction score thresholds (edges whose STRING interaction scores exceed the threshold are retained).

Score dataset	Specht	Khan	Leduc-TMT32	Leduc-plexDIA
150	0.2506/**0.2909**	0.2366/**0.3299**	0.2584/**0.2988**	0.2030/**0.2337**
400	0.4035/**0.4831**	0.4044/**0.4763**	0.3655/**0.4328**	0.3688/**0.4393**
700	0.5754/**0.6201**	0.5925/**0.6350**	0.5613/**0.6166**	0.4970/**0.5424**
900	0.6648/**0.6831**	0.6680/**0.6984**	0.6334/**0.6632**	0.5902/**0.6389**

Values are reported as “without scYOU / with scYOU,” where “without scYOU” denotes curated complex recovery using the original STRING PPI network, and “with scYOU” denotes curated complex recovery after scYOU embedding-guided PPI refinement. Bold values indicate results obtained with scYOU embedding-guided PPI refinement.

The same trend is observed for accuracy (Table 9), where embedding-guided pruning improves ACC at every threshold for all datasets. The gains are most pronounced at lower thresholds (e.g. score 150: 0.2506 to 0.2909 on Specht, 0.2366 to 0.3299 on Khan), and remain stable as the threshold increases (e.g. score 900: 0.6648 to 0.6831 on Specht and 0.6680 to 0.6984 on Khan). Together, these results indicate that scYOU embeddings provide an informative similarity prior that can filter out a subset of unreliable STRING interactions, thereby increasing the signal-to-noise ratio of the reconstructed PPI network and improving downstream recovery of curated complex-associated organization.

This conclusion is further supported by the relative improvement analysis in Fig. 6. Across both similarity measures (PCC and Euclidean) and both settings (with and without incorporating STRING as the base network), embedding integration leads to performance improvements for both ClusterONE and PC2P under ACC and F1. The magnitude of improvement varies across datasets, with Leduc-plexDIA showing particularly large variance and occasional strong gains, while Specht and Khan exhibit more stable yet consistent improvements. Overall, the concordant evidence from Tables 8–9 and Fig. 6 demonstrates that scYOU-derived embeddings can effectively guide interaction refinement and translate into measurable performance gains in curated complex recovery.

**Figure 6:**
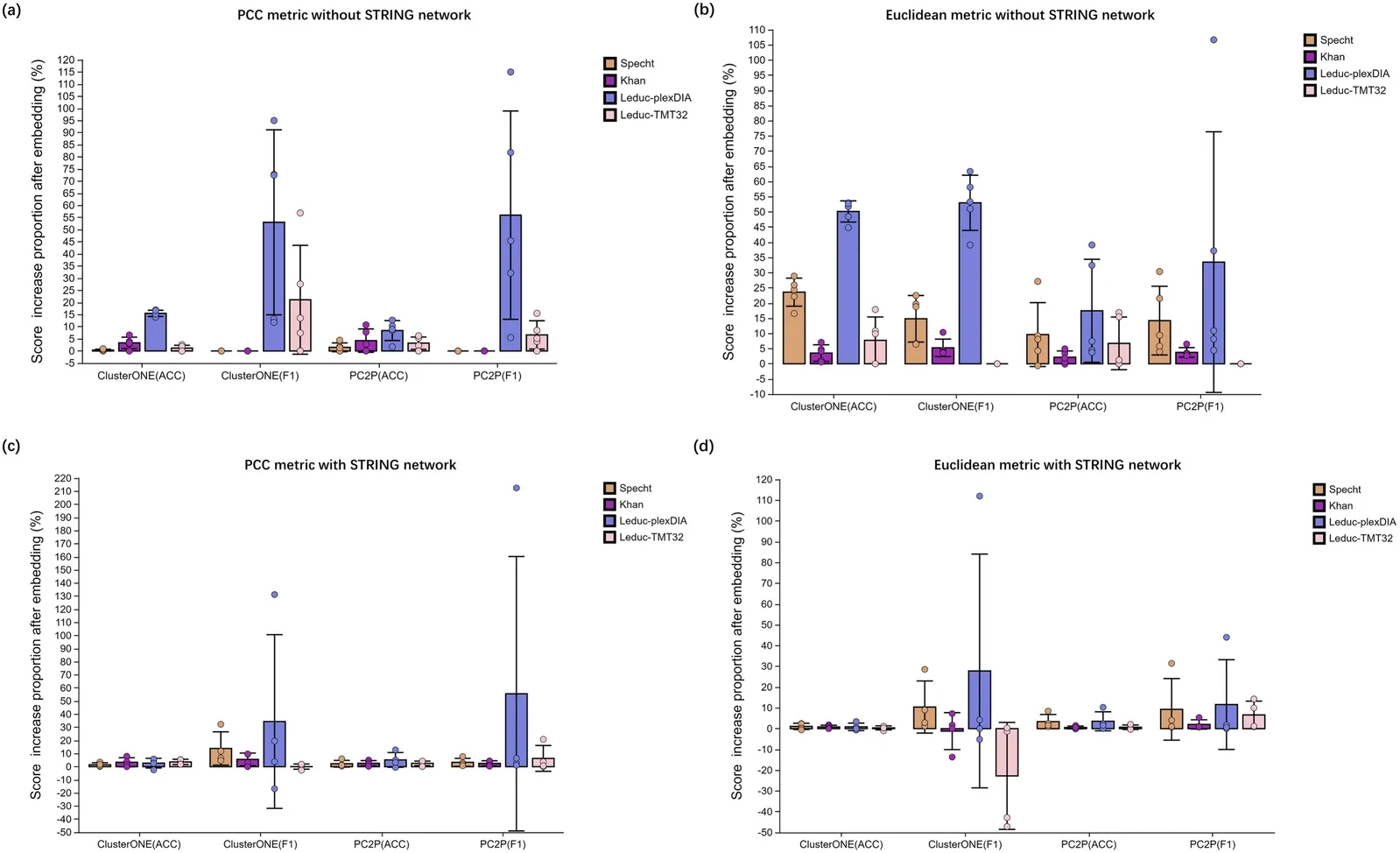
Relative improvement in curated complex recovery after incorporating scYOU embeddings, showing percentage changes in ACC and F1 for ClusterONE and PC2P across four datasets (Specht, Khan, Leduc-TMT32, and Leduc-plexDIA), with comparisons between PCC- and Euclidean-based similarity measures and network settings with and without STRING.

Extended ablation analyses further showed that the improvement in curated complex recovery could not be attributed solely to direct GO similarity or expression-derived co-abundance. The default CC-regularized protein representation provided the most consistent overall performance, while alternative GO branches did not yield a systematic advantage across datasets and STRING confidence levels. Detailed comparisons are provided in Supplementary Note S1 and the corresponding Supplementary Tables.

### Ablation study reveals the contributions of supercell construction and alignment

To disentangle the contributions of cross-channel alignment and supercell-guided structure to cell clustering, we evaluated five ablation variants. **noAlign-noSc** removes both supercell construction and alignment; **noAlign-Sc** retains supercell construction but removes alignment; **Align-noSc-cell** introduces same-cell cross-channel alignment without supercells; **Align-noSc-random** uses randomly generated pseudo-labels to define alignment pairs; and **Align-Sc (Ours)** uses expression-structure-informed supercell assignments to construct cross-channel positive pairs.

All variants were evaluated over a separate set of ten paired random seeds, with identical seeds shared across variants and independent of those used in the main clustering benchmark. Results are reported as mean *± * standard deviation in Table 10 and Fig. 7. Statistical differences were assessed using the Friedman test followed by paired Wilcoxon signed-rank tests comparing Align-Sc with each ablation variant and Holm correction for multiple comparisons; complete statistical results are provided in Supplementary Table S19A–S19B.

**Table 10:** Multi-seed ablation study of supercell construction and alignment.

Module	Metric	Dataset					
		Montalvo	Specht	Khan	Huffman	Leduc-plexDIA	Leduc-TMT32
**noAlign-noSc**	ARI	0.3277*± *0.0410	0.2779*± *0.1498	**0.2367*± *0.0166**	0.8940*± *0.0516	0.9975*± *0.0080	0.5692*± *0.0060
	NMI	0.5873*± *0.0269	0.3458*± *0.0686	0.3283*± *0.0133	0.8254*± *0.0677	**0.9965*± *0.0112**	0.6482*± *0.0110
**noAlign-Sc**	ARI	0.3017*± *0.0114	0.2798*± *0.0972	0.2301*± *0.0157	**0.9101*± *0.0404**	0.9753*± *0.0000	0.5585*± *0.0072
	NMI	0.5581*± *0.0067	0.3553*± *0.0487	0.3236*± *0.0147	**0.8480*± *0.0566**	0.9647*± *0.0000	0.6299*± *0.0062
**Align-noSc-cell**	ARI	**0.8097*± *0.0221**	0.2844*± *0.0486	0.2292*± *0.0148	0.8598*± *0.2089	0.9459*± *0.0832	**0.8430*± *0.0941**
	NMI	0.7499*± *0.0139	0.3599*± *0.0257	0.3288*± *0.0109	0.8033*± *0.2134	0.9463*± *0.0803	**0.8229*± *0.0904**
**Align-noSc-random**	ARI	0.8013*± *0.0237	0.3111*± *0.0488	0.2275*± *0.0150	0.8109*± *0.2933	0.9476*± *0.0765	0.7822*± *0.1419
	NMI	0.7477*± *0.0122	0.3736*± *0.0259	0.3263*± *0.0136	0.7591*± *0.2771	0.9453*± *0.0745	0.7698*± *0.1236
**Align-Sc (Ours)**	ARI	0.8041*± *0.0223	**0.3254*± *0.1231**	0.2311*± *0.0156	0.9078*± *0.0297	**0.9986*± *0.0005**	0.8087*± *0.1131
	NMI	**0.7521*± *0.0110**	**0.3783*± *0.0712**	**0.3296*± *0.0125**	0.8457*± *0.0397	**0.9971*± *0.0014**	0.7929*± *0.0963

ARI and NMI are reported as mean *± * standard deviation over ten paired random seeds. The best mean result for each dataset is highlighted in bold, and the second-best result is underlined.

**Figure 7:**
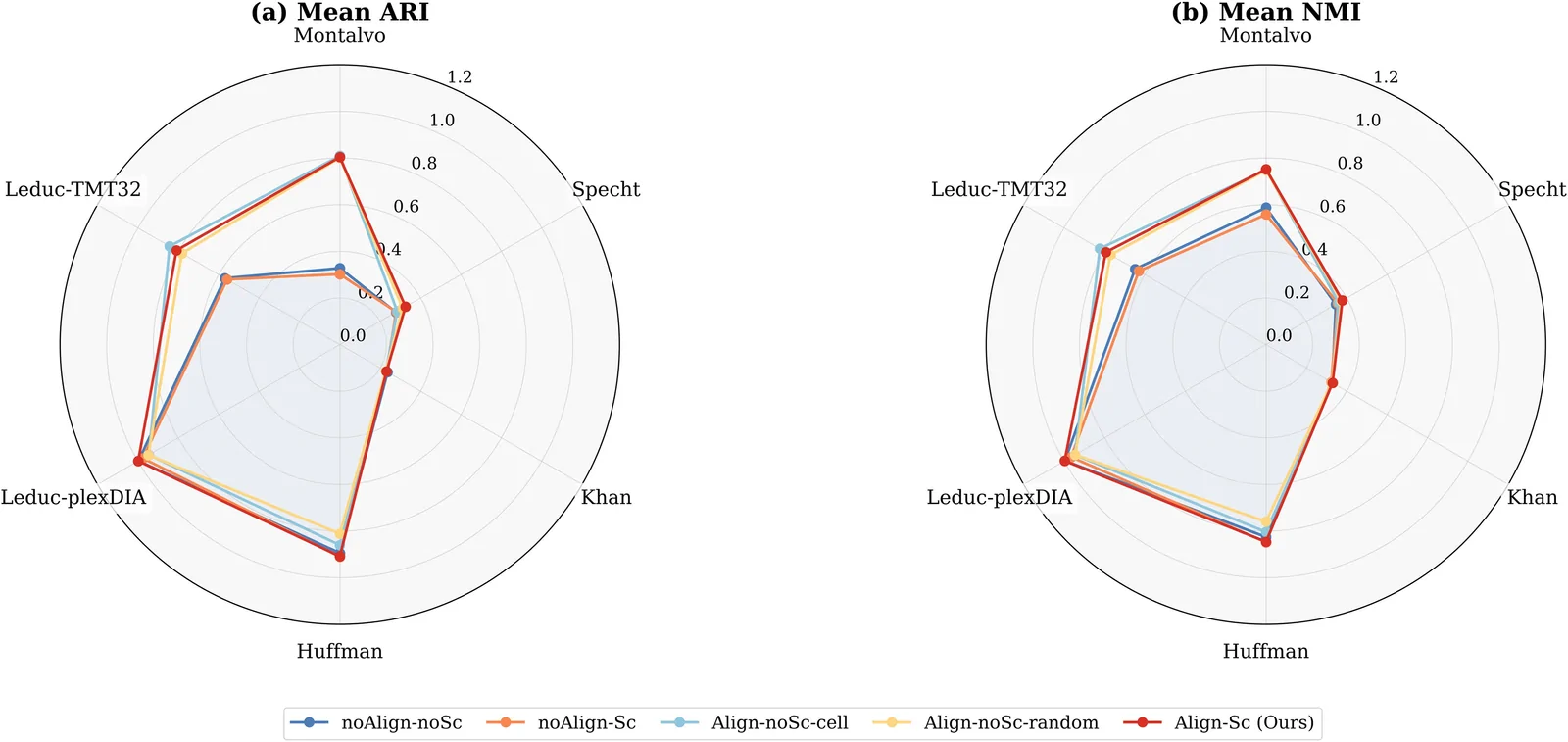
Radar plots of the multi-seed ablation results across six datasets. (a) Mean ARI and (b) mean NMI obtained by different ablation variants over ten paired random seeds. Each axis corresponds to one dataset, and higher values indicate better clustering performance.

Across ten paired random seeds, the ablation variants showed dataset-dependent performance, whereas the complete Align-Sc configuration provided the most consistent overall behavior across datasets. Align-Sc ranked first or second in mean performance for all 12 dataset–metric combinations. Significant improvements over the no-alignment variants were observed on several datasets, including Montalvo, Leduc-TMT32, and Leduc-plexDIA (Supplementary Table S19A–S19B).

Cell-wise and random pseudo-label alignment also achieved competitive performance on individual datasets, indicating that general cross-channel regularization contributes to representation learning. Nevertheless, Align-Sc achieved higher mean ARI and NMI than Align-noSc-random across all six datasets. Although these pairwise differences were not individually significant after Holm correction, their consistent direction supports expression-structure-informed supercell assignments as a more stable structured constraint than random alignment. On the Khan dataset, Align-Sc achieved the highest mean NMI (0.3296*± *0.0125) and the second-highest mean ARI (0.2311*± *0.0156). Its differences from noAlign-noSc were not statistically significant for either metric, indicating comparable performance between these configurations on this dataset rather than a consistent advantage of the full model.

Overall, these results support complementary but dataset-dependent contributions of cross-channel alignment and supercell-guided structure, with Align-Sc providing the most consistent cross-dataset configuration. Additional controls examining non-singleton-only supercell alignment, continued alignment during refinement, parameter-matched single-channel architectures, and removal of GO-informed protein regularization are provided in Supplementary Note S1 and the corresponding Supplementary Tables.

## Discussion

Additional robustness and sensitivity analyses of stochastic supercell construction, alternative alignment formulations, and model hyperparameters are provided in the Supplementary Note S1. These analyses generally supported the adopted default configuration, while also revealing dataset-dependent sensitivity for several design choices. Notably, the original hard supercell alignment showed more consistent cross-dataset performance than the confidence-weighted soft alternative.

### Methodological limitations and future directions

The current supercell construction is primarily computation-driven, relying on expression similarity and graph connectivity, and therefore does not necessarily correspond to well-defined biological entities. In addition, spatial information is not explicitly incorporated. Future extensions could introduce biological priors, such as marker proteins, pathway activities, lineage information, interaction modules, or spatial neighborhoods, to improve the biological coherence and interpretability of supercells.

The current framework also relies on PCC for similarity estimation and uses the CC branch of GO to regularize protein embeddings. These choices provide a simple and stable formulation, but may not fully capture nonlinear expression relationships or broader biological information beyond subcellular localization. Future work will investigate alternative similarity measures and incorporate BP and MF information to provide more comprehensive constraints on protein representations.

Finally, the relative weighting of reconstruction and alignment objectives is determined using a heuristic scheme based on dataset size. Although this setting provides stable performance across the evaluated datasets, adaptive multi-objective optimization, such as dynamic loss reweighting or learnable weighting parameters, may provide greater flexibility across datasets with different noise levels and degrees of cellular heterogeneity.

## Conclusion

We present scYOU, a multi-scale yoked optimization framework for single-cell proteomics that jointly models cellular heterogeneity and protein-level functional organization within a unified dual-channel architecture. By integrating supercell-guided alignment with GO-based functional regularization, scYOU couples cell-level organization and protein functional similarity to learn coherent and biologically meaningful representations. The supercell construction strategy provides adaptive cross-channel alignment units by retaining singleton cells as individual units while introducing neighborhood-level constraints for locally supported cell groups. GO-informed priors further enhance the functional consistency of protein embeddings.

Across diverse datasets and clustering settings, scYOU demonstrates strong and relatively consistent clustering performance together with improved biological interpretability, supporting cell-type identification and embedding-guided recovery of complex-associated protein modules. Importantly, the framework remains stable under varying hyperparameter settings, underscoring its practical applicability to heterogeneous proteomics data. The multi-seed ablation analysis supported the contribution of dual-channel alignment, suggesting that cell- and protein-scale representations provide complementary information, while supercell-guided alignment yielded a consistently competitive cross-dataset configuration despite dataset-dependent differences in its contribution.

Together, these results highlight the value of structurally coupled multi-level representation learning for single-cell proteomics. By bridging cellular heterogeneity and protein functional organization within a unified modeling paradigm, scYOU provides a scalable foundation for downstream biological analysis and offers a principled framework for integrating functional priors into representation learning in complex omics data.

**Key Points**

- scYOU introduces a multi-scale yoked optimization framework for unsupervised single-cell proteomics analysis, jointly addressing cellular heterogeneity characterization and protein functional organization discovery.
- scYOU couples cell-level and protein-level representation learning through a dual-channel architecture, integrating supercell-constrained alignment for structured cell embeddings and GO-CC-derived priors for biologically consistent protein embeddings.
- Benchmarking across diverse single-cell proteomics datasets demonstrates that scYOU achieves strong and relatively consistent clustering performance together with improved embedding separability, while supporting downstream embedding-guided recovery of complex-associated protein modules.

## Supplementary Material

TableS10_Dimension_Ablation_Summary_bbag626

## Data Availability

The source code and processed datasets are available through the scYOU GitHub repository at https://github.com/anchor-hue/scYou and the associated Zenodo archive at https://zenodo.org/records/18756874. Additional benchmarking results and analysis data are provided in the Supplementary Material.
