# Supplementary material for: scYOU: a multi-scale yoked optimization and unsupervised framework for analysis of cellular heterogeneity and complex-associated protein module recovery
Source: Brief Bioinform. 2026 Nov 9;27(6):bbag626. doi: 10.1093/bib/bbag626 (PMC13747026; doi:10.1093/bib/bbag626)
Supplement: Supplementary file 1 [file tables10_dimension_ablation_summary_bbag626.zip › Supplementary_Note_S1_bib_1021.pdf]

Supplementary Material for "scYOU: a multi-scale Yoked Optimization and Unsupervised framework for analysis of cellular heterogeneity and complex-associated protein module recovery"

1 Supplementary Note S1: Extended ablation, robustness, and sensitivity analyses

1.1 Extended ablation analyses of the cell-level architecture

To complement the core ablation analysis presented in Section 3.6 of the main manuscript, we performed additional experiments to further examine specific aspects of the scYOU architecture and training strategy. These analyses evaluate (i) the contribution of non-singleton supercell correspondences, (ii) whether alignment should remain active during task-specific refinement, (iii) whether the performance advantage of scYOU can be explained by increased model capacity, and (iv) whether GO-informed protein regularization contributes directly to cell clustering.

1.1.1 Contribution of non-singleton supercell alignment

Because a substantial fraction of cells are retained as singleton supercells, we further isolated the contribution of genuine multi-cell supercell alignment. We introduced an Align-Sc-multiCell variant in which the original supercell construction was retained, but the alignment loss was computed only for non-singleton supercells ($|S| \geq 2$), with self-pairs excluded so that all positive correspondences represented distinct cells within the same supercell. Singleton cells continued to participate in reconstruction and clustering but contributed no alignment pairs. This variant was compared with same-cell alignment (Align-noSc-cell) and the complete Align-Sc configuration using the same separate set of ten paired random seeds as the preceding ablation analysis, independently of the seed set used in the main clustering benchmark (Supplementary Table S20).

The multi-cell-only variant showed lower mean ARI and NMI than the complete Align-Sc configuration on all six datasets. Compared with same-cell alignment (Align-noSc-cell), it showed lower mean ARI and NMI on five of the six datasets, while achieving higher mean values on Leduc-plexDIA. The consistent advantage of the complete Align-Sc configuration over Align-Sc-multiCell indicates that restricting alignment exclusively to genuine multi-cell pairs is not sufficient to reproduce the performance of the complete model. These results further indicate that the effectiveness

of the full supercell strategy does not arise from genuine multi-cell alignment alone. Instead, the results support an adaptive correspondence scheme in which singleton supercells preserve identity-level alignment, while non-singleton supercells introduce additional neighborhood-level cross-cell constraints where supported by the local expression structure.

1.1.2 Effect of retaining alignment during refinement

We further examined whether cross-channel alignment should remain active during task-specific refinement. In this variant, the alignment loss was retained together with the cell clustering and protein GO objectives throughout refinement, while all other model settings were kept unchanged. The comparison was repeated over ten matched random seeds for each dataset (Supplementary Table S21).

Retaining alignment during refinement produced a clear dataset-dependent effect rather than a consistent improvement. On Montalvo, continued alignment improved ARI from (0.8153 ± 0.0219) to (0.8710 ± 0.0312) and NMI from (0.7517 ± 0.0198) to (0.7809 ± 0.0180), with both improvements remaining significant after Holm correction. In contrast, substantial reductions were observed on Specht and Huffman. For example, ARI decreased from (0.3603 ± 0.0418) to (0.2121 ± 0.1066) on Specht and from (0.9366 ± 0.0110) to (0.6147 ± 0.3132) on Huffman, with corresponding paired differences significant after multiple-testing correction. Performance on Khan and Leduc-plexDIA showed no significant improvement from retaining alignment.

Continued alignment also increased sensitivity to random initialization on some datasets. Most notably, the standard deviation of ARI on Leduc-TMT32 increased from (0.0279) under the default staged training strategy to (0.2918) when alignment was retained, reflecting highly heterogeneous outcomes across seeds. Taken together, these results indicate that persistent cross-channel coupling can be beneficial in individual settings but does not provide a consistent cross-dataset advantage. The default training strategy therefore uses alignment to establish structured cross-channel correspondence during pretraining and subsequently allows the cell and protein channels to undergo task-specific refinement, providing a more stable compromise across heterogeneous single-cell proteomics datasets.

1.1.3 Comparison with parameter-matched single-channel controls

To further determine whether the performance advantage of the dual-channel architecture could be explained simply by its larger model capacity, we compared scYOU with two additional single-channel controls. Cell-only retains only the cell-oriented encoder, decoder, reconstruction objective, and clustering objective, while removing the protein channel and cross-channel alignment. Cell-only-PM uses the same single-channel objective but increases only the hidden-layer widths to closely match the number of trainable parameters of the full scYOU model, while retaining the same 32-dimensional latent representation. The hidden dimensions were determined solely according to parameter count rather than clustering performance. Across all six datasets, the relative parameter-count difference between Cell-only-PM and scYOU was below 0.003%. Both single-channel controls were evaluated over ten paired random seeds using the same preprocessing, clustering, and training settings as scYOU. Detailed parameter

counts and statistical comparisons are provided in Supplementary Tables S22A and S22B, respectively.

Table S1 Comparison with single-channel and parameter-matched controls. ARI and NMI are reported as mean ± standard deviation over ten paired random seeds. Cell-only-PM retains a single cell-oriented representation pathway while closely matching the trainable parameter count of the full scYOU model. The best mean result for each dataset and metric is highlighted in bold, and the second-best is underlined.

Model	Metric	Dataset					
		Montalvo	Specht	Khan	Huffman	Leduc-plexDIA	Leduc-TMT32
Cell-only	ARI	0.3285±0.0419	0.2811±0.1461	0.2375±0.0142	0.8950±0.0508	**0.9975±0.0080**	0.6109±0.1328
	NMI	0.5881±0.0304	0.3471±0.0678	0.3270±0.0158	0.8269±0.0667	0.9965±0.0112	0.6811±0.1051
Cell-only-PM	ARI	0.2929±0.0030	0.2290±0.0462	0.2337±0.0169	0.9314±0.0157	0.9672±0.0634	0.5657±0.0033
	NMI	0.5869±0.0074	0.3275±0.0265	0.3265±0.0145	0.8768±0.0248	0.9666±0.0626	0.6458±0.0039
**scYOU**	ARI	**0.8153±0.0219**	**0.3603±0.0418**	**0.2389±0.0075**	**0.9366±0.0110**	0.9898±0.0132	**0.8856±0.0279**
	NMI	**0.7517±0.0198**	**0.4003±0.0243**	**0.3333±0.0044**	**0.8858±0.0149**	0.9877±0.0098	**0.8625±0.0248**

As shown in Table S1, scYOU achieved higher mean ARI and NMI than the parameter-matched Cell-only-PM model on all six datasets, covering all 12 dataset–metric comparisons. The improvements over Cell-only-PM were statistically significant for both ARI and NMI on Montalvo, Specht, and Leduc-TMT32 after Holm correction. Importantly, increasing the capacity of the single-channel model alone did not produce a systematic performance gain: Cell-only-PM outperformed the original Cell-only model only on Huffman, while showing lower mean performance in the remaining 10 of 12 comparisons. These results indicate that the advantage of scYOU cannot be explained solely by its larger parameter count and support the benefit of modeling the same expression matrix from complementary cell-oriented and protein-oriented perspectives.

1.1.4 Effect of GO-informed protein regularization on cell clustering

To directly assess whether GO-informed regularization of the protein channel contributes to cell clustering within the dual-channel framework, we introduced an Align-Sc-noGO control. This variant retains the complete dual-channel architecture, supercell-guided alignment, reconstruction and clustering objectives, and all other training settings, while removing only the GO regularization term by setting $\delta = 0$. The comparison was performed over ten paired random seeds on all six datasets.

Removing GO-informed regularization did not measurably alter cell clustering performance: Align-Sc-noGO produced the same mean ARI and NMI as the complete Align-Sc configuration across all six datasets under the evaluated settings (Supplementary Table S23). This result indicates that the clustering performance of the dual-channel framework is not driven by the GO prior. This observation is consistent with the staged optimization strategy, in which GO regularization is applied during protein-channel refinement after cross-channel alignment has been deactivated. Thus, under the default training configuration, GO primarily serves as a protein-side biological constraint rather than an additional source of cell-clustering supervision.

1.2 Extended ablation analyses of protein-level representation and PPI refinement

To further determine which components of the learned protein representation contribute to the recovery of curated protein complexes, we performed an extended ablation study of embedding-guided PPI pruning. Starting from STRING-derived PPI networks filtered at different interaction score thresholds, we compared direct similarity-based pruning with a series of protein-embedding variants that differed in the Gene Ontology (GO) information used during representation learning.

The comparison included ten settings. STRING only denotes the original STRING network without additional pruning. For GO-only similarity pruning, protein-pair similarity was calculated directly from GO semantic similarity without learning protein embeddings. For expression-only similarity pruning, similarity was calculated directly from the original protein abundance profiles across cells. These two settings therefore test whether either functional annotation or co-abundance alone is sufficient to improve the STRING network.

We next evaluated whether representation learning itself provides additional information beyond these direct similarity measures. Embedding-guided pruning denotes protein embeddings learned by the dual-channel architecture without GO regularization. We further trained otherwise identical variants using different GO branches as protein-level priors, including BP alone, MF alone, BP+CC, MF+CC, and BP+MF+CC. The default scYOU embedding-guided pruning uses only the Cellular Component (CC) branch during training. Thus, this extended comparison evaluates not only whether learned protein embeddings improve PPI refinement, but also whether the choice of GO ontology branch affects downstream recovery of curated complexes.

For all pruning-based settings, the same pruning ratio was used within each dataset and STRING interaction-score threshold to ensure a controlled comparison. ClusterONE was subsequently applied to each resulting network, and performance was evaluated using the same F1-score and accuracy metrics as in the main experiment.

The detailed results of the similarity-source ablation experiments are summarized in Tables S2 and S3. The results first show that neither GO semantic similarity nor expression-derived co-abundance alone fully accounts for the benefit of scYOU. Direct GO-only pruning improved over the original STRING network in many settings and was particularly effective for Leduc-TMT32 at the lower STRING thresholds and for Specht at the threshold of 700. Expression-only pruning also produced improvements in several settings, but its effects were less consistent. For example, at the STRING threshold of 700, expression-only pruning reduced both F1-score and accuracy on Specht relative to the original STRING network. These observations indicate that functional annotation and co-abundance each contain useful information for PPI refinement, but neither provides a uniformly reliable surrogate for complex-associated organization.

The comparison between direct similarity measures and learned embeddings further demonstrates the contribution of representation learning. Even without GO regularization, embedding-guided pruning generally improved upon direct expression-based similarity, particularly on Khan and Leduc-plexDIA. This suggests that the

Table S2 Ablation study of similarity sources for embedding-guided PPI pruning using F1-score by ClusterONE.

Score	Method	Specht	Khan	Leduc-TMT32	Leduc-plexDIA
150	STRING only	0.0238	0.0247	0.0000	0.0097
	GO-only similarity pruning	0.0306	0.0584	**0.0508**	0.0145
	Expression-only similarity pruning	0.0359	0.0867	0.0186	0.0198
	Embedding-guided pruning	0.0375	0.1103	0.0257	0.0146
	Embedding-guided pruning (BP)	0.0382	0.1149	0.0286	0.0236
	Embedding-guided pruning (MF)	0.0380	0.1068	0.0301	0.0250
	Embedding-guided pruning (BP + CC)	0.0415	0.1201	0.0295	0.0233
	Embedding-guided pruning (MF + CC)	0.0423	0.1332	0.0328	**0.0289**
	Embedding-guided pruning (BP + MF + CC)	0.0394	0.1405	0.0307	0.0264
	scYOU embedding-guided pruning	**0.0447**	**0.1480**	0.0349	0.0271
400	STRING only	0.1238	0.1333	0.0656	0.1218
	GO-only similarity pruning	0.1842	0.1517	**0.1569**	0.1362
	Expression-only similarity pruning	0.1686	0.1745	0.0895	0.1498
	Embedding-guided pruning	0.1937	0.1803	0.0921	0.1518
	Embedding-guided pruning (BP)	0.2014	0.1828	0.0939	0.1632
	Embedding-guided pruning (MF)	0.1944	0.1873	0.1021	0.1674
	Embedding-guided pruning (BP + CC)	0.2145	0.2004	0.0962	0.1621
	Embedding-guided pruning (MF + CC)	0.2257	0.1832	0.1147	0.1693
	Embedding-guided pruning (BP + MF + CC)	0.2179	0.1925	0.0982	0.1622
	scYOU embedding-guided pruning	**0.2419**	**0.2083**	0.1089	**0.1704**
700	STRING only	0.4541	0.5530	0.3043	0.2967
	GO-only similarity pruning	**0.5993**	0.5964	0.4295	0.3516
	Expression-only similarity pruning	0.4218	0.5487	0.3668	0.3492
	Embedding-guided pruning	0.4596	0.6480	0.0349	0.3524
	Embedding-guided pruning (BP)	0.4872	0.6552	0.4038	0.3605
	Embedding-guided pruning (MF)	0.4830	0.6491	0.4252	0.3659
	Embedding-guided pruning (BP + CC)	0.5117	0.6753	0.4076	0.3781
	Embedding-guided pruning (MF + CC)	0.5364	0.6631	0.4689	0.3744
	Embedding-guided pruning (BP + MF + CC)	0.5528	0.6906	0.5184	0.3723
	scYOU embedding-guided pruning	0.5776	**0.7215**	**0.5506**	**0.3927**
900	STRING only	0.6956	0.7510	**0.6102**	0.5571
	GO-only similarity pruning	0.7013	0.7709	0.6034	0.5943
	Expression-only similarity pruning	0.7072	0.7604	0.6068	0.5736
	Embedding-guided pruning	0.6942	0.7645	0.5944	0.6052
	Embedding-guided pruning (BP)	0.6810	0.7693	0.6071	0.6077
	Embedding-guided pruning (MF)	0.6863	0.7802	0.5936	0.5985
	Embedding-guided pruning (BP + CC)	0.6957	0.7736	0.5728	0.5938
	Embedding-guided pruning (MF + CC)	0.7095	0.7825	0.5870	0.6024
	Embedding-guided pruning (BP + MF + CC)	0.7081	0.7800	0.5921	0.6178
	scYOU embedding-guided pruning	**0.7154**	**0.7846**	0.6005	**0.6248**

protein channel captures higher-order structure from the abundance matrix that is not preserved by pairwise co-abundance similarity alone. Introducing GO-derived regularization further altered the organization of the protein embedding space, with the effect depending on the ontology branch used.

Importantly, the extended GO-branch ablation provides a direct rationale for using GO-CC as the default prior in scYOU. The default CC-regularized scYOU representation outperformed the otherwise identical embedding model without GO regularization in all 16 dataset-threshold combinations for F1-score and in 14 of 16 combinations for accuracy. In contrast, replacing CC with BP or MF, or combining BP and MF with CC, did not yield a consistent additional improvement. Although several alternative configurations performed best in individual settings—for example, MF+CC at a STRING threshold of 150 on Leduc-plexDIA and GO-only similarity on Leduc-TMT32 at lower thresholds—no alternative GO configuration showed a systematic advantage across datasets and network-confidence levels.

Across all ten evaluated settings, the default scYOU embedding-guided pruning achieved the highest F1-score in 11 of the 16 dataset-threshold combinations. GO-only pruning was best in three combinations, MF+CC embedding regularization in one,

Table S3 Ablation study of similarity sources for embedding-guided PPI pruning using accuracy by ClusterONE.

Score	Method	Specht	Khan	Leduc-TMT32	Leduc-plexDIA
150	STRING only	0.2506	0.2366	0.2584	0.2030
	GO-only similarity pruning	0.2628	0.2687	**0.3295**	0.2118
	Expression-only similarity pruning	0.2736	0.2942	0.2807	0.2226
	Embedding-guided pruning	0.2859	0.3137	0.2634	0.2315
	Embedding-guided pruning (BP)	0.2830	0.3156	0.2684	0.2307
	Embedding-guided pruning (MF)	0.2814	0.3048	0.2701	0.2296
	Embedding-guided pruning (BP + CC)	0.2876	0.3090	0.2886	0.2315
	Embedding-guided pruning (MF + CC)	**0.3006**	0.2847	0.1949	0.2236
	Embedding-guided pruning (BP + MF + CC)	0.2897	0.3268	0.2940	0.2208
	scYOU embedding-guided pruning	0.2909	**0.3299**	0.2988	**0.2337**
400	STRING only	0.4035	0.4044	0.3655	0.3688
	GO-only similarity pruning	0.4596	0.4258	0.4269	0.3895
	Expression-only similarity pruning	0.4241	0.4496	0.4075	0.4142
	Embedding-guided pruning	0.4481	0.4595	**0.4616**	0.4068
	Embedding-guided pruning (BP)	0.4569	0.4728	0.3923	0.4276
	Embedding-guided pruning (MF)	0.4330	0.4653	0.4155	0.4239
	Embedding-guided pruning (BP + CC)	0.4514	0.4570	0.4038	0.4305
	Embedding-guided pruning (MF + CC)	0.4726	0.4636	0.3981	0.4347
	Embedding-guided pruning (BP + MF + CC)	0.4329	0.4628	0.4146	0.4370
	scYOU embedding-guided pruning	**0.4831**	**0.4763**	0.4328	**0.4393**
700	STRING only	0.5754	0.5925	0.5613	0.4970
	GO-only similarity pruning	**0.6697**	0.6166	0.5981	0.5219
	Expression-only similarity pruning	0.5448	0.5899	0.5763	0.5173
	Embedding-guided pruning	0.5871	0.5932	0.6042	0.5290
	Embedding-guided pruning (BP)	0.5538	0.5950	0.6073	0.5306
	Embedding-guided pruning (MF)	0.6346	0.6002	0.5834	0.5294
	Embedding-guided pruning (BP + CC)	0.6219	0.6213	0.6018	0.5247
	Embedding-guided pruning (MF + CC)	0.5742	0.6188	0.6147	0.5417
	Embedding-guided pruning (BP + MF + CC)	0.5523	0.6235	0.5951	0.5362
	scYOU embedding-guided pruning	0.6201	**0.6350**	**0.6166**	**0.5424**
900	STRING only	0.6648	0.6680	0.6334	0.5902
	GO-only similarity pruning	0.6705	0.6774	0.6428	0.6265
	Expression-only similarity pruning	0.6761	0.6886	0.6537	0.6016
	Embedding-guided pruning	0.6925	0.6935	0.6083	0.6138
	Embedding-guided pruning (BP)	0.6840	0.6723	0.6535	0.6270
	Embedding-guided pruning (MF)	0.6883	0.6581	0.6517	0.6059
	Embedding-guided pruning (BP + CC)	0.6901	0.6720	0.6248	0.6273
	Embedding-guided pruning (MF + CC)	**0.7038**	0.6633	0.6616	0.6357
	Embedding-guided pruning (BP + MF + CC)	0.6915	0.6418	0.6439	0.6325
	scYOU embedding-guided pruning	0.6831	**0.6984**	**0.6632**	**0.6389**

and the unmodified STRING network in one. A similar pattern was observed for accuracy: scYOU achieved the highest value in 11 of 16 combinations, while the remaining best-performing settings were distributed among GO-only pruning, MF+CC regularization, and the embedding model without GO regularization. Thus, the advantage of scYOU does not arise from universally dominating every individual condition, but from providing the most consistent performance across datasets and STRING confidence levels.

These results also indicate that broader GO information is not necessarily more informative for the present task. BP and MF describe biological processes and molecular activities that may be shared by proteins participating in related pathways without requiring them to belong to the same molecular assembly. In contrast, CC more directly encodes subcellular localization and annotated cellular structures. The observation that adding BP and/or MF to CC did not consistently improve performance therefore supports our use of CC as the primary GO prior in the final scYOU model, while also showing that the contribution of individual ontology branches can vary across datasets.

The effect of PPI pruning additionally depended on the confidence of the underlying STRING network. At low and intermediate STRING thresholds, where the

starting network contains a larger number of potentially weak or noisy interactions, embedding-guided refinement generally produced larger improvements. At the strictest threshold of 900, the differences among methods became smaller. In particular, on Leduc-TMT32, the original STRING network achieved the highest F1-score (0.6102), whereas scYOU obtained 0.6005. This result suggests that when the initial network is already highly stringent, additional pruning may remove a small number of informative edges. Nevertheless, scYOU achieved the highest accuracy on the same dataset at this threshold (0.6632), indicating that the refined network retained favorable overall agreement with the curated reference complexes.

Taken together, these ablation results show that scYOU protein embeddings cannot be reduced to direct GO similarity or expression co-abundance. Their performance reflects the combined contribution of learned abundance-derived structure, dual-channel optimization, and GO-CC-based protein regularization. At the same time, the occasional advantage of GO-only or alternative ontology configurations highlights the dataset dependence of protein-network refinement and argues against interpreting any single source of functional similarity as direct evidence of physical complex formation.

1.3 Robustness to stochastic supercell pruning

Because Bernoulli-based pruning introduces randomness into supercell construction, we further assessed whether this stochasticity affects both the final clustering performance and the resulting supercell graph structure. To this end, we repeated the supercell construction step using five different pruning seeds and then ran scYOU with fixed model initialization and identical training settings. We evaluated ARI and NMI and additionally quantified key supercell statistics, including the number of constructed supercells, supercell size distribution, and the fraction of singleton cells.

Table S4 Robustness of scYOU to stochastic supercell pruning across random seeds (Mean ± Standard Deviation over 5 Runs).

Metrics / Datasets	Montalvo	Specht	Khan	Huffman	Leduc-plexDIA	Leduc-TMT32
ARI	**0.7903**±0.0069	**0.3383**±0.0149	**0.1882**±0.0019	**0.9177**±0.0150	**0.9951**±0.0099	**0.8914**±0.0140
NMI	**0.7542**±0.0055	**0.3894**±0.0087	**0.3121**±0.0029	**0.8608**±0.0222	**0.9929**±0.0141	**0.8767**±0.0112

Table S5 Supercell construction statistics across datasets under stochastic pruning. Values are reported as mean ± standard deviation over five random seeds. Singleton cell fraction denotes the fraction of original cells that remain as singleton supercells after pruning.

Dataset	#Cells	#Supercells	Mean size	Median size	Max size	Singleton fraction
Montalvo	843	734.20±5.72	1.15±0.01	1.00±0.00	16.00±1.87	0.826±0.008
Specht	1490	1258.20±7.56	1.18±0.01	1.00±0.00	28.00±3.46	0.798±0.005
Khan	1408	1128.20±3.83	1.25±0.00	1.00±0.00	16.00±2.35	0.719±0.003
Huffman	373	326.80±3.70	1.14±0.01	1.00±0.00	10.40±1.52	0.810±0.011
Leduc-plexDIA	121	72.00±3.94	1.68±0.10	1.00±0.00	16.60±2.19	0.534±0.035
Leduc-TMT32	1526	682.80±11.56	2.24±0.04	1.00±0.00	9.00±0.00	0.316±0.008

As summarized in Table S4, the variation across random seeds remains limited on all datasets. The standard deviations of ARI range from 0.0019 to 0.0150, while those of NMI range from 0.0029 to 0.0222, which are small relative to their corresponding mean scores. These results indicate that the stochastic pruning procedure introduces only minor variability into the final clustering outcomes.

We further examined whether stochastic pruning leads to unstable or dataset-specific supercell graph construction. As shown in Table S5, the number of generated supercells is highly stable across random seeds for all datasets. For example, the standard deviation of the number of supercells ranges from 3.70 on Huffman to 11.56 on Leduc-TMT32, which is small relative to the total number of cells and supercells. The supercell size distribution also remains stable across runs, with median supercell size consistently equal to 1 and small variations in mean supercell size. This indicates that the pruning procedure produces a reproducible supercell structure rather than highly variable graph partitions.

The fraction of singleton cells varies across datasets, ranging from 0.316 on Leduc-TMT32 to 0.826 on Montalvo. This dataset-dependent variation reflects differences in local cell-cell similarity structure and graph connectivity after pruning. Accordingly, the proportion of cells participating in non-singleton supercells ranges from 0.174 on Montalvo to 0.684 on Leduc-TMT32. The high singleton fraction on several datasets indicates that supercell construction should not be interpreted as uniformly aggregating cells for denoising. Rather, it provides an adaptive local grouping mechanism: cells without sufficiently supported neighbors retain identity-level correspondence, whereas non-singleton groups introduce cross-cell constraints where stronger local structure is available. Consistent with this interpretation, the non-singleton-only alignment experiment showed that restricting alignment exclusively to genuine multi-cell pairs yielded lower mean ARI and NMI than the complete Align-Sc configuration across all six datasets. Relative to same-cell alignment, the non-singleton-only variant showed lower mean performance on five datasets but higher mean ARI and NMI on Leduc-plexDIA (Supplementary Table S20). These results suggest that the complete supercell strategy benefits from retaining identity-level correspondence for singleton cells in addition to selectively introducing multi-cell constraints for non-singleton supercells, while the relative performance of same-cell and multi-cell-only alignment remains dataset dependent. Importantly, however, stable clustering performance is observed across repeated runs despite these differences in singleton fraction and supercell size distribution. These results suggest that the performance of scYOU is not driven by an unstable dataset-specific graph construction artifact, but rather remains robust to the stochasticity introduced during supercell pruning.

1.4 Sensitivity to supercell construction and alignment formulation

1.4.1 Sensitivity to supercell construction parameters

To further assess the impact of the hyperparameters a (neighborhood size) and b (shared neighbor threshold) on cell clustering performance during supercell construction, we performed a sensitivity analysis by systematically varying their values (Fig. S1–S2).

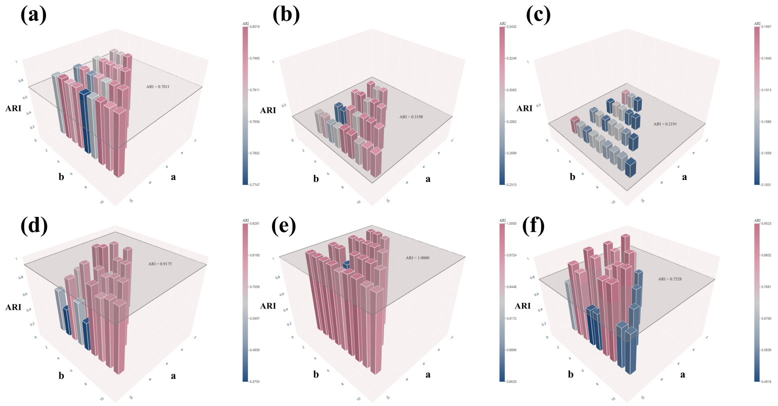

Fig. S1 Sensitivity of cell clustering performance (ARI) to supercell construction hyperparameters. (a–f) Three-dimensional bar plots of ARI values as functions of the neighborhood size a and the shared neighbor threshold b on the Montalvo, Specht, Khan, Huffman, Leduc-plexDIA, and Leduc-TMT32 datasets, respectively. The light gray horizontal plane in each subpanel indicates the ARI achieved by the best-performing state-of-the-art baseline on the corresponding dataset, serving as a reference for performance comparison under different hyperparameter settings.

Referring to Figs. S1–S2, we observe that the influence of the supercell construction parameters a and b is dataset dependent. For the Montalvo, Specht, Khan, and Leduc-plexDIA datasets, both ARI and NMI show relatively limited variation over the examined parameter combinations, whereas more pronounced fluctuations are observed for Huffman and Leduc-TMT32. Nevertheless, clustering performance remains competitive over a broad range of parameter settings. These results indicate that the supercell construction is generally effective across different parameter choices, while also revealing increased sensitivity on a subset of datasets.

1.4.2 Comparison of hard and confidence-weighted soft supercell alignment

To further examine whether replacing the discrete Bernoulli-pruned supercell assignment with a confidence-aware coupling could improve the alignment, we additionally evaluated a confidence-weighted soft alignment variant. Instead of using the binary supercell assignment after Bernoulli pruning, the soft variant retains the connected-component structure before pruning and directly uses the retention probability RP_i as

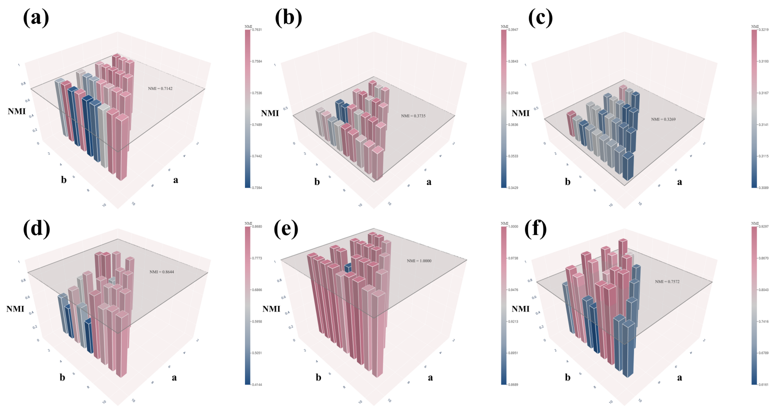

Fig. S2 Sensitivity of cell clustering performance (NMI) to supercell construction hyperparameters. (a–f) Three-dimensional bar plots of NMI values as functions of the neighborhood size a and the shared neighbor threshold b on the Montalvo, Specht, Khan, Huffman, Leduc-plexDIA, and Leduc-TMT32 datasets, respectively. The light gray horizontal plane in each subpanel indicates the NMI achieved by the best-performing state-of-the-art baseline on the corresponding dataset, serving as a reference for performance comparison under different hyperparameter settings.

the structural confidence of cell i. Let C_i denote the pre-pruning connected-component assignment. The pairwise alignment weight is defined as

$$w_{ij} = \begin{cases} 1, & i = j, \\ \sqrt{RP_i RP_j}, & i \neq j \text{ and } C_i = C_j, \\ 0, & C_i \neq C_j. \end{cases} \tag{1}$$

Accordingly, replacing the binary same-supercell relation defined in the main manuscript with w_{ij} gives

$$\mathcal{L}_{\text{align}}^{\text{soft}} = -\frac{1}{n}\sum_{i=1}^{n} \log \left(\frac{\sum_{j=1}^{n} w_{ij} \exp\left(\kappa((\hat{\mathbf{H}}_{\text{cell}}^{(K)})^i, ((\hat{\mathbf{H}}_{\text{protein}}^{(K)})^\top)^j)/\tau\right)}{\sum_{j=1}^{n} \exp\left(\kappa((\hat{\mathbf{H}}_{\text{cell}}^{(K)})^i, ((\hat{\mathbf{H}}_{\text{protein}}^{(K)})^\top)^j)/\tau\right)} \right). \tag{2}$$

We compared the original hard alignment with this soft variant using the same five random seeds for both formulations. As shown in Table S6, hard alignment consistently achieved higher mean ARI and NMI than soft alignment across all six datasets. Averaged across the six datasets, hard alignment achieved an ARI of 0.6977 and an NMI of 0.6924, compared with 0.3895 and 0.4434, respectively, for soft alignment. The performance gap was particularly pronounced on Montalvo, Specht, Huffman, and Leduc-TMT32, whereas the two formulations were relatively close on Khan and Leduc-plexDIA.

These results indicate that continuously propagating lower-confidence supercell relations does not provide a consistent benefit for scYOU and can substantially weaken clustering performance on several datasets. In contrast, the original hard formulation provides a stronger structural filtering effect by restricting alignment to the retained supercell relations. We therefore retained the original hard supercell alignment as the

Table S6 Comparison of hard and confidence-weighted soft supercell alignment. Both variants were evaluated using the same five random seeds, and results are reported as mean ± standard deviation. The higher mean value for each metric on each dataset is highlighted in bold.

Dataset	Hard ARI	Soft ARI	Hard NMI	Soft NMI
Montalvo	**0.8091±0.0146**	0.3636±0.0863	**0.7303±0.0397**	0.6103±0.0452
Specht	**0.3411±0.0315**	0.0613±0.1002	**0.3752±0.0196**	0.0781±0.0698
Khan	**0.2226±0.0163**	0.2098±0.0174	**0.3178±0.0179**	0.3115±0.0171
Huffman	**0.9061±0.0490**	0.4035±0.2499	**0.8465±0.0661**	0.3514±0.2351
Leduc-plexDIA	**0.9951±0.0110**	0.9853±0.0218	**0.9929±0.0158**	0.9809±0.0276
Leduc-TMT32	**0.9121±0.0439**	0.3132±0.2260	**0.8915±0.0478**	0.3282±0.2664
Average	**0.6977**	0.3895	**0.6924**	0.4434

default formulation of scYOU, while reporting the soft formulation as an additional sensitivity analysis.

1.5 Sensitivity to model configuration and training hyperparameters

We further evaluated the sensitivity of scYOU to key model configurations and training hyperparameters, including the reconstruction weight α, alignment weight β, clustering weight γ, GO regularization weight δ, temperature parameter τ, and latent embedding dimensionality d. For the loss-related hyperparameter analyses, one hyperparameter was varied while all others were fixed to their default values. The reconstruction weight α was set according to dataset size, with $\alpha = 1$ for smaller datasets and $\alpha = 0.5$ for larger datasets. For the dimensionality analysis, the cell- and protein-channel embedding dimensions were jointly varied over $d \in \{16, 32, 64, 128\}$ while all other model and training settings were kept unchanged. A summary of the tested ranges and main observations is provided in Table S7. The complete loss-related hyperparameter results are reported in Supplementary Table S9, and the complete latent-dimensionality results are provided in Supplementary Table S10.

Table S7 Summary of model configuration and training hyperparameter sensitivity analysis. Representative values cover alternative latent dimensions, term removal or weakening, default settings, and stronger regularization. Complete loss-related hyperparameter results are provided in Supplementary Table S9, and complete latent-dimensionality results are provided in Supplementary Table S10.

Parameter	Role	Default	Representative values	Main observation
α	Reconstruction weight	1.0 or 0.5	0.1, 0.5, 1.0, 2.0	Moderate values worked better; extreme values degraded clustering.
β	Alignment weight	0.1	0.0, 0.1, 0.5, 1.0	Small alignment was beneficial; large weights reduced ARI/NMI.
γ	Clustering weight	1.0	0.0, 1.0, 5.0	Removing this term degraded several datasets; default-scale values were stable.
δ	GO regularization weight	1.0	0.0, 1.0, 5.0	Limited influence under current loss scaling.
τ	Temperature	0.5	0.1, 0.5, 1.0, 2.0	Dataset-dependent sensitivity; 0.5 was a competitive default.
d	Latent dimension	32	16, 32, 64, 128	$d = 32$ achieved the highest mean ARI and NMI on all six datasets.

Among the loss-related hyperparameters, scYOU was most sensitive to α, β, and τ. Moderate reconstruction and alignment weights generally led to better clustering performance, whereas overly large weights often degraded the results. The clustering weight γ was important for learning discriminative cell representations, as removing this term reduced performance in several datasets. In contrast, δ had limited effect on the cell clustering metrics under the current scaling, consistent with GO regularization acting primarily on the protein channel rather than directly optimizing cell clustering. We additionally examined the sensitivity of scYOU to latent embedding dimensionality by jointly setting the cell- and protein-channel dimensions to $d \in \{16, 32, 64, 128\}$. Each configuration was evaluated over ten independent runs on all six datasets while all other settings were kept unchanged. Overall, $d = 32$ showed the most consistent cross-dataset performance, achieving the highest mean ARI and NMI on all six datasets (Supplementary Table S10). In addition, the $d = 32$ configuration exhibited the lowest run-to-run standard deviation for both ARI and NMI on all six datasets among the tested dimensions. These results support $d = 32$ as a well-supported default latent dimensionality for both channels. Taken together, these sensitivity analyses support the adopted configuration as a consistent cross-dataset setting rather than a parameterization optimized for a single dataset. In particular, $d = 32$ was retained as the default latent dimensionality for both channels because it achieved the highest mean ARI and NMI while exhibiting the lowest run-to-run variability across all six datasets among the tested dimensions.

Supplementary Figure Legends

Figure S1. Sensitivity of cell clustering performance (ARI) to supercell construction hyperparameters. (a–f) Three-dimensional bar plots of ARI values as functions of the neighborhood size a and the shared neighbor threshold b on the Montalvo, Specht, Khan, Huffman, Leduc-plexDIA, and Leduc-TMT32 datasets, respectively. The light gray horizontal plane in each subpanel indicates the ARI achieved by the best-performing state-of-the-art baseline on the corresponding dataset, serving as a reference for performance comparison under different hyperparameter settings.

Figure S2. Sensitivity of cell clustering performance (NMI) to supercell construction hyperparameters. (a–f) Three-dimensional bar plots of NMI values as functions of the neighborhood size a and the shared neighbor threshold b on the Montalvo, Specht, Khan, Huffman, Leduc-plexDIA, and Leduc-TMT32 datasets, respectively. The light gray horizontal plane in each subpanel indicates the NMI achieved by the best-performing state-of-the-art baseline on the corresponding dataset, serving as a reference for performance comparison under different hyperparameter settings.

Figure S3. t-SNE visualization of cell representations on the Montalvo dataset. t-SNE projections of the processed expression features and representations obtained using competing methods and scYOU. Points are colored according to the annotated cell types.

Figure S4. t-SNE visualization of cell representations on the Khan dataset. t-SNE projections of the processed expression features and representations obtained using competing methods and scYOU. Points are colored according to the annotated cell types.

Figure S5. t-SNE visualization of cell representations on the Huffman dataset. t-SNE projections of the processed expression features and representations obtained using competing methods and scYOU. Points are colored according to the annotated cell types.

Figure S6. t-SNE visualization of cell representations on the Leduc-plexDIA dataset. t-SNE projections of the processed expression features and representations obtained using competing methods and scYOU. Points are colored according to the annotated cell types.

Figure S7. t-SNE visualization of cell representations on the Leduc-TMT32 dataset. t-SNE projections of the processed expression features and representations obtained using competing methods and scYOU. Points are colored according to the annotated cell types.
